# Cryo-EM structures of the ATP-bound Vps4^E233Q^ hexamer and its complex with Vta1 at near-atomic resolution

**DOI:** 10.1038/ncomms16064

**Published:** 2017-07-17

**Authors:** Shan Sun, Lin Li, Fan Yang, Xiaojing Wang, Fenghui Fan, Mengyi Yang, Chunlai Chen, Xueming Li, Hong-Wei Wang, Sen-Fang Sui

**Affiliations:** 1 State Key Laboratory of Membrane Biology, Beijing Advanced Innovation Center for Structural Biology, School of Life Sciences, Tsinghua University, Beijing 100084, China; 2 School of Life Sciences, Tsinghua-Peking Joint Center for Life Sciences, Beijing Advanced Innovation Center for Structural Biology, Tsinghua University, Beijing 100084, China; 3 Ministry of Education Key Laboratory of Protein Science, Beijing Advanced Innovation Center for Structural Biology, Tsinghua-Peking Joint Center for Life Sciences, School of Life Sciences, Tsinghua University, Beijing 100084, China

## Abstract

The cellular ESCRT-III (endosomal sorting complex required for transport-III) and Vps4 (vacuolar protein sorting 4) comprise a common machinery that mediates a variety of membrane remodelling events. Vps4 is essential for the machinery function by using the energy from ATP hydrolysis to disassemble the ESCRT-III polymer into individual proteins. Here, we report the structures of the ATP-bound Vps4^E233Q^ hexamer and its complex with the cofactor Vta1 (vps twenty associated 1) at resolutions of 3.9 and 4.2 Å, respectively, determined by electron cryo-microscopy. Six Vps4^E233Q^ subunits in both assemblies exhibit a spiral-shaped ring-like arrangement. Locating at the periphery of the hexameric ring, Vta1 dimer bridges two adjacent Vps4 subunits by two different interaction modes to promote the formation of the active Vps4 hexamer during ESCRT-III filament disassembly. The structural findings, together with the structure-guided biochemical and single-molecule analyses, provide important insights into the process of the ESCRT-III polymer disassembly by Vps4.

## Abstract (web summary)

The ESCRT-III and Vps4 complexes mediate a variety of membrane remodelling events. Here the authors describe the structure of the Vps4 hexamer complexed to its cofactor Vta1, and show that Vta1 bridges adjacent Vps4 subunits to promote formation of the active hexamer during ESCRT-III filament disassembly.

The ESCRT-III (endosomal sorting complex required for transport -III) machinery and the Vps4 (vacuolar protein sorting 4) contribute to the events of membrane budding away from the cytosol^1^,^2^. They play essential roles in diverse cellular processes, including the multivesicular body biogenesis, the extracellular vesicle formation, enveloped virus budding, midbody abscission during cytokinesis, wound plasma membrane repair, neural pruning, nuclear pore complex assembly and nuclear envelope reformation^2^,^3^. The dynamic assembly and disassembly cycle of ESCRT-III proteins between monomeric states in the cytosol and heteropolymeric states on the membrane is critical for the efficient ESCRT-III function^4^,^5^.

The disassembly of ESCRT-III is mainly mediated by the Vps4 protein that is a member of the type I AAA+ ATPase (ATPase associated with various cellular activities) family and oligomerizes into a functional hexamer with elevated ATPase activity upon binding to ATP^6^. Powered by ATP hydrolysis, the Vps4 oligomer, promoted by the cofactor protein Vta1 (Vps twenty associated 1), disassembles ESCRT-III polymers into individual ESCRT-III subunits available for recycling^6^,^7^,^8^,^9^,^10^. Vps4 protein contains an MIT (microtubule interacting and trafficking) domain at the N terminus, a single ATPase cassette comprising a large ATPase domain and a small ATPase domain with a β-domain inserted within it, and a C-terminal helix^8^,^11^,^12^,^13^ (Fig. 1a, upper panel). The structure of the ATPase cassette resembles those from other AAA+ ATPase members^8^,^11^,^12^,^13^, except for the β-domain that is responsible for the binding to Vta1 (refs 8, 14). Vta1 promotion of Vps4 function *in vivo* and ATPase activity *in vitro*^9^,^15^,^16^,^17^,^18^ is largely determined by Vta1 structural features. The N-terminal domain (NTD) of Vta1 consists of two MIT motifs, important for the binding to ESCRT-III subunits Vps60 and Did2 (ref. 19). A flexible linker connects the NTD with the C-terminal domain (CTD) composed of a pair of antiparallel α-helices mediating Vta1 dimerization and Vps4 binding^19^ (Fig. 1a, lower panel).

During the past decade, numerous crystal structures of Vps4 have been reported^8^,^11^,^12^,^13^,^20^,^21^,^22^. The overall structure is composed of a disordered MIT domain, an AAA ATPase cassette, an independent antiparallel β-strand-containing domain inserted within the small domain of the AAA ATPase cassette and a C-terminal helix packing against the large ATPase domain^8^,^11^,^12^,^13^,^20^. The structure of the ATPase cassette of Vps4 is similar to those from other AAA+ ATPase members, except for the β-domain^8^,^11^,^12^,^13^. As expected, the nucleotide binds in a site between the large and small ATPase domains^8^,^11^,^12^,^13^. Crystal structural studies reveal significant structural flexibility within the Vps4 molecule, displayed as *en bloc* rotations of up to 20° between the large and small ATPase domains, whereas little conformational changes within the individual domains, and that nucleotides binding may stabilize a relatively closed conformation^8^,^11^,^12^,^13^,^23^. Notably, only nucleotide-bound forms of SKD1 can be well superimposed onto the hexameric ring structures of nucleotide-bound p97 without any major steric clashes, whereas apo form of SKD1 showed less compatible interfaces between protomers, and this may explain why the apo form of SKD1 is mainly a monomer in solution^13^.

Besides the reported crystal structures of Vps4 (refs 8, 11, 12, 13, 14, 19, 20, 21, 22, 24, 25), the electron microscopy structures of Vps4 in dodecamer or tetradecamer were also obtained claiming that the Vps4 oligomer is a two stacked ring structure with 12 or 14 subunits^20^,^24^,^25^, but these structures appeared to contradict a biochemical analysis that showed that the functional form of Vps4 is hexameric^21^. Although a recent study provided a crystal structure of the Vps4 hexameric ring, the used protein is a Vps4 homologue (MsVps4) from *Metallosphaera sedula*, a prokaryotic organism that lacks endomembrane structures^22^. Moreover, this Vps4 hexamer was obtained in the absence of ATP that is completely different from the eukaryotic Vps4 hexamer whose assembly is ATP dependent. Thus, the structure of this MsVps4 hexamer cannot represent the structure of the active form of the eukaryotic Vps4 hexamer.

In this study, using Vps4 from yeast, we solved the structures of the ATP-bound Vps4^E233Q^ hexamer at 3.9 Å resolution and its complex with Vta1 at 4.2 Å resolution, both determined by single-particle electron cryo-microscopy (cryo-EM) without any symmetry imposed. The near-atomic resolution structures reveal rich details for the arrangement of the Vps4^E233Q^ subunits in the hexamer, the interactions between Vps4^E233Q^ subunits and between Vps4^E233Q^ and Vta1. The structural findings, together with structure-guided biochemical and single-molecule fluorescence analyses, not only explain the assemblies of the Vta1-free Vps4 hexamer and the Vta1-bound Vps4 hexamer but also reveal the mechanisms of the ATP-dependent oligomerization of Vps4 and the Vta1-promoting activity on Vps4. Our results provide important insights into the disassembly process of the ESCRT-III polymer by the Vps4–Vta1 complex.

## Results

### Structures of ATP-bound Vps4 hexamer and Vps4–Vta1 complex

**Figure 1:**
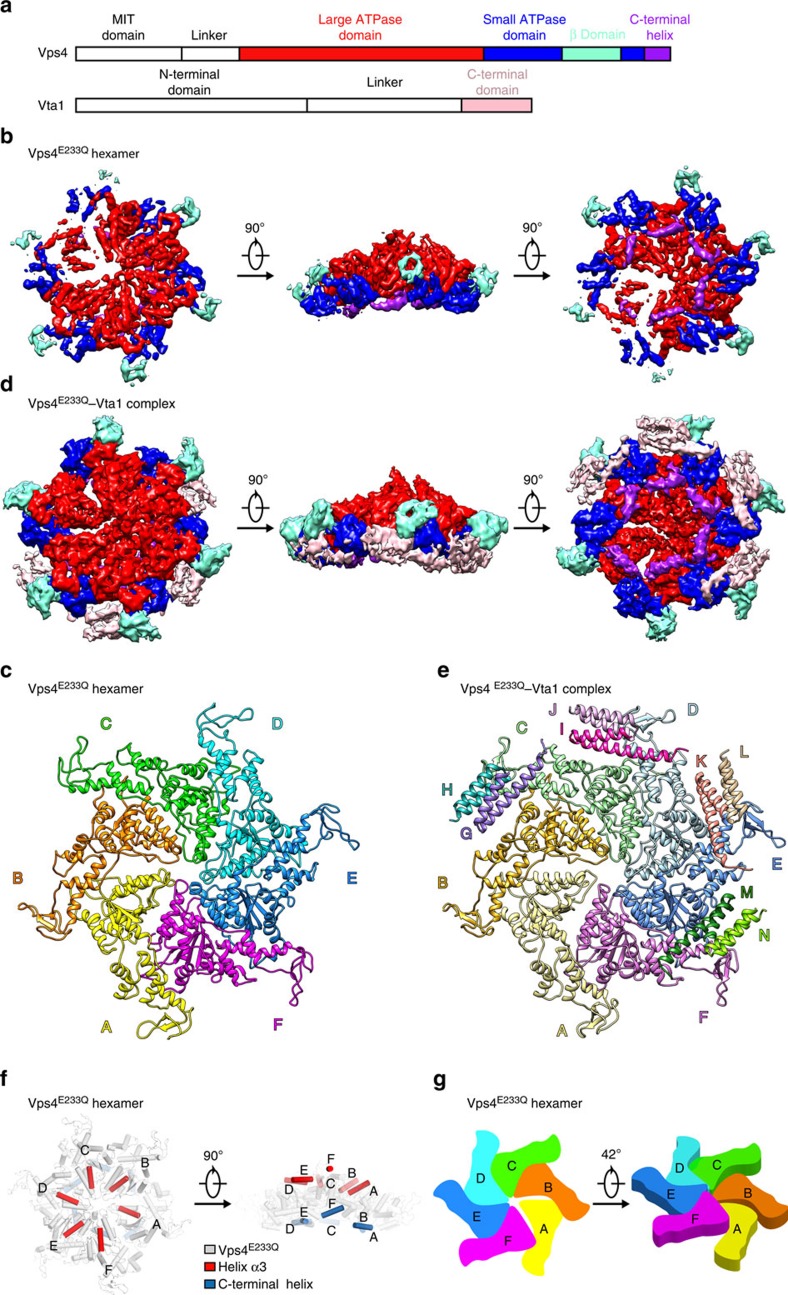
Overall structures of the ATP-bound Vps4^E233Q^ hexamer and its complex with Vta1. (**a**) Domain diagrams of the yeast Vps4 (upper) and Vta1 (lower). (**b**) Different views of the EM density map of the ATP-bound Vps4^E233Q^ hexamer at 5.8 σ contour level. (**c**) The atomic model of the Vps4^E233Q^ hexamer. Vps4^E233Q^ subunits are colour coded. (**d**) Different views of the EM density map of the ATP-bound Vps4^E233Q^–Vta1 complex at the 3.9 σ contour level showing the positions of the Vta1. (**e**) The atomic model of the ATP-bound Vps4^E233Q^–ta1 complex. Vps4^E233Q^ subunits and Vta1 subunits are colour coded. (**f**) Different views of the helix α3 and C-terminal helix in the Vps4^E233Q^ hexamer to show the spiral arrangement of Vps4 subunits. (**g**) A schematic diagram showing the topology of the Vps4^E233Q^ subunits in the Vps4^E233Q^ hexamer. The colouring scheme is the same as in (**c**).

We purified the full-length yeast Vps4 containing an E233Q mutation, which allows ATP binding but blocks ATP hydrolysis, and assembled Vps4^E233Q^ oligomer in the presence of 1 mM ATP *in vitro* (Methods). The assembled Vps4^E233Q^ appeared as a homogenous oligomer with a molecular weight of ∼300 kDa, corresponding to a hexamer, as determined by size exclusion chromatography (SEC) with multiangle light scattering (MALS) (Supplementary Fig. 1a). We used single-particle cryo-EM method to obtain the three-dimensional (3D) reconstruction of the 300 kDa oligomer without imposing any symmetry at a resolution of 3.9 Å (Fig. 1b and Supplementary Fig. 1b–g). The near-atomic resolution of the EM map revealed clear major secondary structural elements with some bulky side chains identifiable (Supplementary Fig. 1e). The structure appeared as a hexameric ring, consistent with the MALS result (Fig. 1b). Although we used the full-length Vps4^E233Q^ for the oligomer assembly and the Vps4^E233Q^ protein in the hexamer was intact as evidenced by the SDS–polyacrylamide gel electrophoresis (SDS–PAGE) gel showing a molecular weight of ∼50 kD (Supplementary Fig. 1a), the six MIT domains exhibit no EM density, likely reflecting their dynamic locations due to the long linker of ∼40 residues between the MIT domain and the followed ATPase cassette (Fig. 1a, upper). Much of the central region of the hexamer including the large and the small ATPase domains were well resolved in the density map (Fig. 1b and Supplementary Fig. 1f). The β-domains located at the periphery were also well identified, although displayed lower resolution (Fig. 1b and Supplementary Fig. 1f). One of the six subunits, subunit A, was not as well resolved in the density map as the other subunits, indicating its potential flexibility (Fig. 1b and Supplementary Fig. 1f).

To investigate the structure detail of the ATP-bound Vps4 hexamer, we built atomic models within the cryo-EM map using the available crystal structure of the ATPγS-bound Vps4^E233Q^ (PDB ID: 3EIH)^12^ as the starting model to refine (Fig. 1c and Supplementary Fig. 1h). Just as shown in the EM map, the hexameric ring structure exhibits a striking asymmetry in subunit juxtaposition (Fig. 1c,f,g and Supplementary Fig. 2a). First, the ring deviates from a sixfold symmetric conformation. The subunit A shifts slightly away from the ring centre (Fig. 1c,f,g), and the interaction interfaces between it and its two adjacent subunits are only about half of those between the other subunits (Supplementary Fig. 2a). This relatively weak interaction of subunit A with its neighbouring subunits may account for its flexibility. Moreover, the ring is not planner, in which subunits arrange in an upward spiral from subunit A to subunit F as manifested by the relative positions of the α3-helix and C-terminal helix in each subunit, resembling a split right-handed spiral ring (Fig. 1f,g). Notably, when superimposing the structures of each subunit, the large ATPase domain of the subunit A exhibits a slightly different position relative to the small ATPase domain as compared with the other five subunits that all perfectly align with each other (Supplementary Fig. 3a). Agreeing with this, densities for nucleotides are clearly visible in the nucleotide-binding pockets of subunit B through F, whereas there is no clear density in the nucleotide-binding pocket of subunit A, suggesting that subunit A is likely free of the nucleotide (Supplementary Fig. 4a). The current resolution limit and the quality of EM maps did not allow conclusive differentiation between ATP and ADP. To verify the nucleotide state in the hexamer, we measured the ATP and ADP levels in the Vta1-bound Vps4^E233Q^ hexamer eluted from the SEC in the nucleotide-free buffer. The result indicates that the ratio of ADP/ATP in the hexamer is 0.036±0.004 (average±s.d.), suggesting that the ADP molecule is almost nonexistent in the hexamer. Meanwhile, given that the protein we used is the Vps4 E233Q mutant that allows ATP binding but blocks ATP hydrolysis and excess ATP molecules were added when we prepared the samples for cryo-EM analysis, the hydrolysis from ATP to ADP is almost impossible. Therefore, all of the above information suggests that the bound nucleotide in the hexamer could be ATP.

To dissect how Vta1 stabilizes the Vps4 oligomer, we performed structural analysis of the ATP-bound Vps4^E233Q^–Vta1complex. Vps4^E233Q^ and Vta1 proteins were mixed and incubated with 1 mM ATP. The preparation was applied to the SEC, and a major peak shifted to a lower elution volume compared with that of the ATP-bound Vps4^E233Q^ hexamer alone or the Vta1 dimer alone was observed. This peak contained both Vps4^E233Q^ and Vta1 as judged by the SDS–PAGE gel, indicating that the two proteins readily formed the stable complex (Supplementary Fig. 5a). We obtained 3D reconstruction of the Vta1-bound Vps4^E233Q^ hexameric complex at a resolution of 4.2 Å (Fig. 1d and Supplementary Fig. 5b–g). The structure of the ATP-bound Vps4^E233Q^–Vta1 complex also contains a hexameric ring of the Vps4^E233Q^ similar to that of the Vta1-free Vps4^E233Q^ hexamer (Fig. 1d and Supplementary Fig. 6a,b). After docking the Vps4^E233Q^ hexamer into the EM map, we can still observe some clear extra densities located between neighbouring Vps4^E233Q^ subunits of subunit B to F distributed at the periphery of the ring (Fig. 1d and Supplementary Fig. 2b). We attributed these densities to the Vta1 protein. Indeed, the crystal dimeric structure of the Vta1 CTD (PDB ID: 2RKL) fits into the densities with high confidence (Supplementary Fig. 2b), consistent with the reported function of the CTD: binding with Vps4 (refs 14, 19). Same as the MIT domain of Vps4, no densities for the NTDs of Vta1 are visible in the map (Fig. 1d), reflecting their dynamic positions due to the very long linker between the NTD and the CTD (Fig. 1a, lower). Notably, the densities for two CTD dimers between Vps4^E233Q^ subunit A and B, and subunit A and F, only show up at very low contour level with low resolution (data not shown). To clarify the poor densities are due to the flexibility or the varied stoichiometry of binding, we investigated the stoichiometry of the ATP-bound Vps4^E233Q^–Vta1 complex using Vps4 and Vta1 proteins labelled by two different fluorescent dyes. The result indicates that the molar ratio of Vta1/Vps4^E233Q^ in the complex is 2.06±0.11 (average±s.d.). We therefore conclude that Vta1 and Vps4^E233Q^ form a complex of ∼2:1 stoichiometry in the presence of ATP. Thus, the poor densities for the Vta1 dimers in the map of the ATP-bound Vps4^E233Q^–Vta1 complex are attributed to the flexibility that is also in agreement with the high flexibility of subunit A in the Vps4 oligomer.

We were able to build an atomic model for the six Vps4^E233Q^ molecules and four Vta1 CTD dimers in the cryo-EM map of the ATP-bound Vps4^E233Q^–Vta1 complex (Fig. 1e and Supplementary Fig. 5h). The overall structure of the Vta1-bound Vps4^E233Q^ hexamer and the Vta1-free Vps4^E233Q^ hexamer share similar conformations with a root mean square deviation (r.m.s.d.) of 2.18 Å overall Cα atoms (Fig. 1c,e). The structure features of the Vta1-free Vps4^E233Q^ hexamer described above, including the loose contact between subunit A and its neighbouring subunits (Supplementary Fig. 2b), the spiral arrangement of subunits from A through F (Supplementary Fig. 6c) and the different conformation of subunit A compared with other subunits (Supplementary Figs 3a,4b), are all preserved in the Vta1-bound Vps4^E233Q^ hexamer, suggesting that Vta1 binding does not, or at least not significantly, alter the conformation of the ATP-bound Vps4^E233Q^ hexamer. The two CTDs in one dimer organize antiparallel to each other forming a four-helix bundle, same as in the crystal structure of the CTD dimer, and the four CTD dimers are well aligned with the r.m.s.d. of 1.4 Å over 96 Cα atoms, indicating the rigidity of the CTD dimer (Supplementary Fig. 3b). The interface of the two CTDs in one CTD dimer is roughly perpendicular to the Vps4^E233Q^ hexameric ring, resulting in one CTD, referred to as inner CTD, closer to the ring centre than the other one, referred to as outer CTD (Fig. 1e and Supplementary Figs 2b,3b). Notably, in our map, the helix α8 of the inner CTD is much longer than that of the outer CTD (Fig. 1e and Supplementary Figs 2b,3b), suggesting that the helix α8 is partially flexible, consistent with the crystal structure observation that the N-terminal part of helix α8 displays great conformational variability in the CTD dimer^19^.

### Characterization of protein interactions in the structures

Our cryo-EM work represents the experimentally determined atomic model of the ATP-bound Vps4^E233Q^ hexamer with or without the cofactor Vta1, allowing visualization of specific interactions governing Vps4 oligomerization in its active state. Since the structure of the Vps4^E233Q^ hexamer present in the Vps4^E233Q^–Vta1 complex is very similar with that in the Vta1-free Vps4^E233Q^ hexamer, we next only investigated the interface in the Vta1-free Vps4^E233Q^ hexamer in detail.

**Figure 2:**
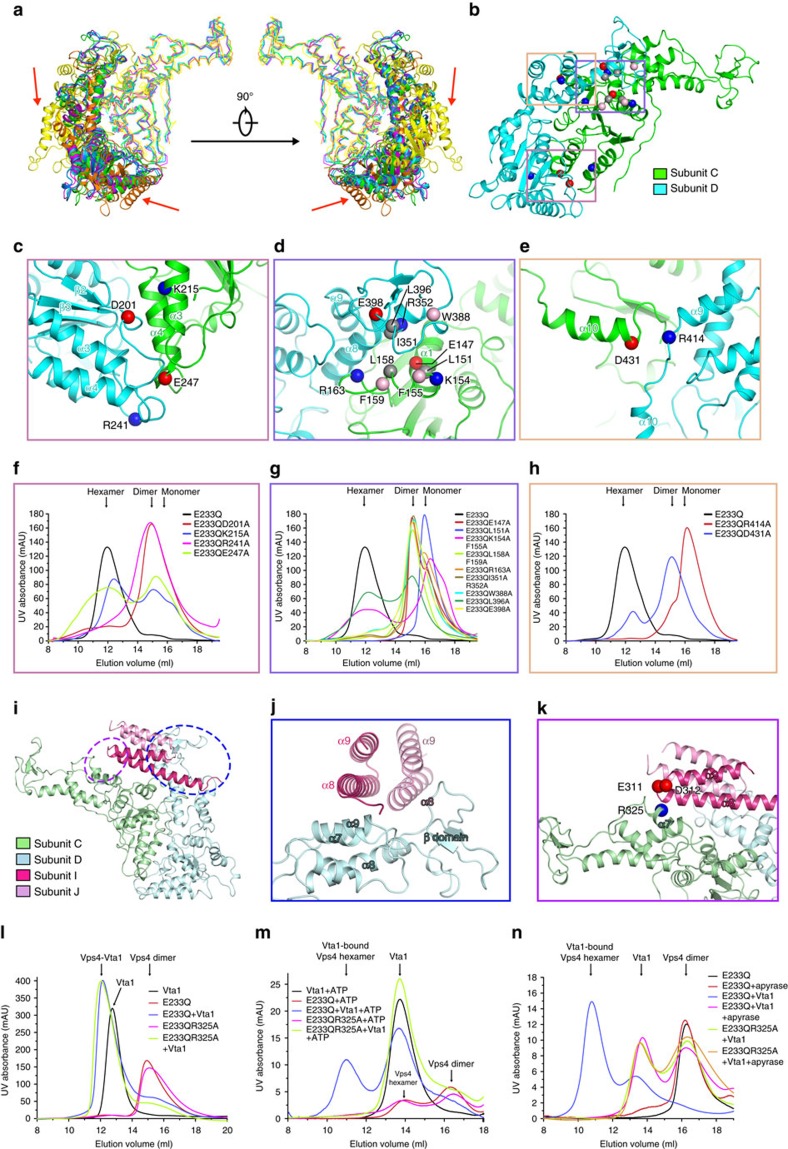
Characterizations of protein interactions in the Vta1-free and the Vta1-bound Vps4^E233Q^ hexamers. (**a**) Comparison of six pairs of the adjacent Vps4^E233Q^ subunits. The colouring scheme is the same as in Fig. 1c,e. When one Vps4^E233Q^ molecule (shown in ribbon representation) from each pair of the adjacent subunits aligned with each other, the other one (shown in cartoon representation) is also well aligned, except for the pairs involving subunit A (red arrow). (**b**) A focused view of the domains that mediate the interactions between the Vps4^E233Q^ monomers (subunits C and D). Three boxed intermonomer interfaces are detailed in **c**–**e**. (**c**–**e**) The close-up views of the interfaces between the large ATPase domains (**c**), the large and small ATPase domains including the β-domain (**d**) and the C-terminal helixes (**e**). Potential residues participated in the interactions are represented by spheres. Hydrophobic, aromatic, positively charged and negatively charged residues are coloured grey, pink, blue and red, respectively. The orientations of all close-up views are optimized to show the interactions. (**f**–**h**) SEC analyses of the oligomeric states of the Vps4^E233Q^ mutants targeting the interface between the large ATPase domains (**f**), the large and small ATPase domains including the β-domain (**g**) and the C-terminal helixes (**h**). (**i**) A focused view of the interactions between the Vta1 CTD dimer (subunits I and J) and the adjacent Vps4^E233Q^ subunit pair (subunits C and D). Two different contact modes are highlighted by purple and blue dashed circles, respectively, and detailed in **j**,**k**. (**j**,**k**) The close-up views of the interactions between the Vps4^E233Q^ subunit D and the CTD dimer (**j**), and the Vps4^E233Q^ subunit C and the CTD dimer (**k**). (**l**,**m**) Assessments of the interactions by SEC between Vta1 and Vps4 mutants, E233Q and E233QR325A, at high concentration (100 μM) in the absence of ATP (**l**), and at low concentration (5 μM) in the presence of ATP (**m**). (**n**) Assessment of the stabilities of the Vta1-free Vps4^E233Q^ hexamer and the Vta1-bound Vps4^E233Q^ hexamer formed by different Vps4 mutants with or without apyrase treatment by SEC (See Methods for details).

When one Vps4^E233Q^ molecule from each pair of the adjacent subunits aligned with each other, the other one is also well aligned, except for the pairs involving subunit A (Fig. 2a). This is expected as the interface areas between subunit A and its neighbouring subunits are much smaller as described above (Supplementary Fig. 2a). Therefore, we mainly focused on the analysis of the four similar interfaces and discussed interface between subunits C and D as a representative.

The inter-subunit interactions occur both between the large ATPase domains and between the large ATPase and the small ATPase domains including the β-domain (Fig. 2b). The large ATPase domain interaction contributed by helix α3 and α4 from one subunit, and the loops between strand β2 and helix α3 and between strand β3 and helix α4 from neighbouring subunit, largely composed of polar residues, indicating that electrostatic or hydrogen-bonding interactions dominate this interface (Fig. 2b,c). The interaction between the large and small ATPase domains including the β-domain involves the E147-R163 region comprising the C-terminal half of helix α1 and the following loop from one subunit, and the C-terminal half of helix α8 and the Trp388-Glu398 region of β-domain from the neighbouring subunit (Fig. 2b,d). Atomic interactions at this interface are versatile, involving polar, hydrophobic and aromatic residues, suggesting a combination of hydrogen bonds, salt bridges and van der Waals contacts (Fig. 2b,d). Additional contact occurs between residues in the C-terminal helix of one subunit and the corresponding amino acids in the linker region between helix α9 and C-terminal helix of the neighbouring subunit, exemplified by potential hydrogen bonds or salt bridges between Asp431 and Arg414 (Fig. 2b,e). To validate the above interfaces and residues involved in oligomerization of Vps4, we constructed Vps4 mutants based on the E233Q mutant: D201A, K215A, R241A and E247 for the large ATPase domain interactions (Fig. 2c); E147A, L151A, K154AF155A, L158AF159A, R163A, I351AR352A, W388A, L396A and E398A for interaction between the large and small ATPase domains including the β-domain (Fig. 2d); and R414A and D431A for the C-terminal helix interactions (Fig. 2e). All of these mutants can be purified but eluted on SEC with eliminated or substantially reduced amount of hexamers in the presence of ATP (Fig. 2f–h). Importantly, all of these residues are highly conserved in eukaryotes, suggesting a conserved mechanism of Vps4 oligomerization (Supplementary Fig. 7a). Previous studies based on the Vps4 hexamer models derived from the p97 hexameric structure and the crystal packing interactions in Vps4 crystal forms have discovered some residues, L151 and R352, essential for the oligomerization^8^,^12^,^21^. These interactions are also observed in our structure and are all at the interface between the large and small ATPase domains (Fig. 2b,d,g). Moreover, our structure discovered that the interactions between the large ATPase domains and between the C-terminal helices are also important for the hexamer assembly (Fig. 2b,c,e,f,h).

To further evaluate the functional relevance of the ATP-bound Vps4^E233Q^ hexamer structure, we mutated subunit interface residues that substantially affect the Vps4^E233Q^ assembly and compared the ATPase activities of these mutants with the wild-type Vps4. As shown in Supplementary Fig. 8, all of the mutants showed severely decreased ATPase activities to 20% of the wild-type activity and most of them reduced to ∼10%, suggesting that the key residues identified in affecting Vps4^E233Q^ assembly based on ATP-bound Vps4^E233Q^ hexamer structure are also important for the ATPase activity of the wild-type Vps4 protein. These results confirmed the functional relevance of the subunit interface discovered by the ATP-bound Vps4^E233Q^ hexamer structure.

We next investigated the structure of the Vta1-bound Vps4^E233Q^ complex to understand the mechanism of Vta1-promoting activity on Vps4. Vta1 CTD dimer bridges between two adjacent Vps4^E233Q^ subunits in two different modes (Fig. 2i). One mode is guided by the interactions of the β-domain and helixes α7 and α9 of the small ATPase domain from one Vps4^E233Q^ subunit with the regions around the tip of α8/α9 helix hairpin from the outer CTD of one CTD dimer and the N-terminal half part of the helix α8 from the inner CTD of the same dimer, respectively (Fig. 2i,j). This interaction is consistent with the crystal structures of the Vta1 VSL dimer in complex with a Vps4 fragment^14^ and the Vta1 CTD dimer^19^, and a model generated by aligning these two crystal structures^26^ as reported previously. The other interaction mode, completely different from that observed before, occurs between the helix α7 of the neighbouring Vps4^E233Q^ subunit and the α8/α9 helix hairpin tip of only the inner CTD, exemplified by potential polar contacts between Arg325 of Vps4, and Glu311 and Asp312 of Vta1 (Fig. 2i,k). The inner CTD binds to both of the adjacent Vps4^E233Q^ subunits simultaneously and hence is more stable than the outer CTD that binds to only one Vps4^E233Q^ subunit (Figs 1e and 2i). Also, the tip of α8/α9 helix hairpin from each CTD in one CTD dimer makes contribution to both of the binding modes by interacting with different regions of the adjacent Vps4^E233Q^ subunits (Fig. 2i–k).

To corroborate the structural observations, we generated Vps4 mutant E233QR325A and examined its ability to associate with Vta1. Similar to the E233Q mutant, the E233QR325A mutant ran as a dimer without ATP and a hexamer in the presence of ATP on SEC, indicating this mutation does not affect the oligomerization ability of Vps4 (Supplementary Fig. 9). In the absence of ATP, both Vps4 mutant constructs can bind to Vta1 via the β-domain only at high concentration due to the weak interaction between Vps4 β-domain and Vta1 VSL as manifested by the relatively high Kd value of 85 μM (Fig. 2l)^14^. When ATP is present, Vps4^E233Q^ assembles into hexamer forming an additional site for Vta1 to bind as revealed by our structure, and therefore should enhance the binding of Vta1. Indeed, in the presence of ATP, Vta1 can form complex with Vps4^E233Q^ hexamer at a concentration as low as 5 μM of Vps4 (Fig. 2m). In contrast, when R325A is introduced to the additional binding site, Vta1 high-binding affinity to Vps4^E233Q^ hexamer is lost (Fig. 2m). Together, these results demonstrated that the interaction between the helix α7 from Vps4^E233Q^ and the tip of α8/α9 helix hairpin from Vta1 is indispensable for efficient binding of Vta1 to the ATP-bound Vps4^E233Q^ hexamer.

We next examined the Vta1 effect on the stability of ATP-induced Vps4^E233Q^ hexamer. We collected the SEC fractions of Vta1-free and Vta1-bound Vps4^E233Q^ hexamers in the presence of ATP and reapplied them to SEC in the absence of ATP. Interestingly, we found that Vta1-free hexamers nearly all disassembled into dimers, whereas the Vta1-bound hexamers remained intact (Fig. 2n). As a control, treatment with apyrase to deplete ATP from the samples before loading to SEC led to full disassembly of both Vta1-free and Vta1-bound hexamers, indicating that ATP still binds to the Vta1-bound hexamer even in a solution lacking ATP once the complex forms (Fig. 2n). These experimental findings strongly argue that Vta1 binding may stabilize the conformation of the ATP-bound Vps4 hexamer, thus facilitating the bound ATP for subsequent hydrolysis. Accordingly, mutation construct E233QR325A of Vps4 hexamer at high concentration incubated with Vta1 disassembled immediately in a solution that lacked ATP (Fig. 2n). Notably, the residue 325 of Vps4 is highly conserved as a positively charged amino acid in eukaryotes, but diverse in crenarchaeota in which the Vta1 is absent, further indicating the importance of this residue for the function of Vta1 (Supplementary Fig. 7b).

The finding of our structure of the Vps4^E233Q^–Vta1 complex is the identification of the second site, R325 of Vps4^E233Q^, for the Vta1 binding. To examine whether this site is also important for the Vta1-promoting activity on wild-type Vps4, we measured the ATPase activity of wild-type and R325A mutant Vps4 in the presence of Vta1. As shown in Supplementary Fig. 8, Vta1 was unable to stimulate the ATPase activity of the R325A mutant, whereas the ATPase activity of the wild-type Vps4 was increased by twofold in the presence of Vta1. As a control, the ATPase activities of the wild-type and the R325A mutant Vps4 proteins alone were similar. These results confirmed the physiological relevance of the second binding site at the Vps4–Vta1 interface even though our structures were obtained using the E233Q mutant Vps4.

### Conformational changes and ATPase active centres

**Figure 3:**
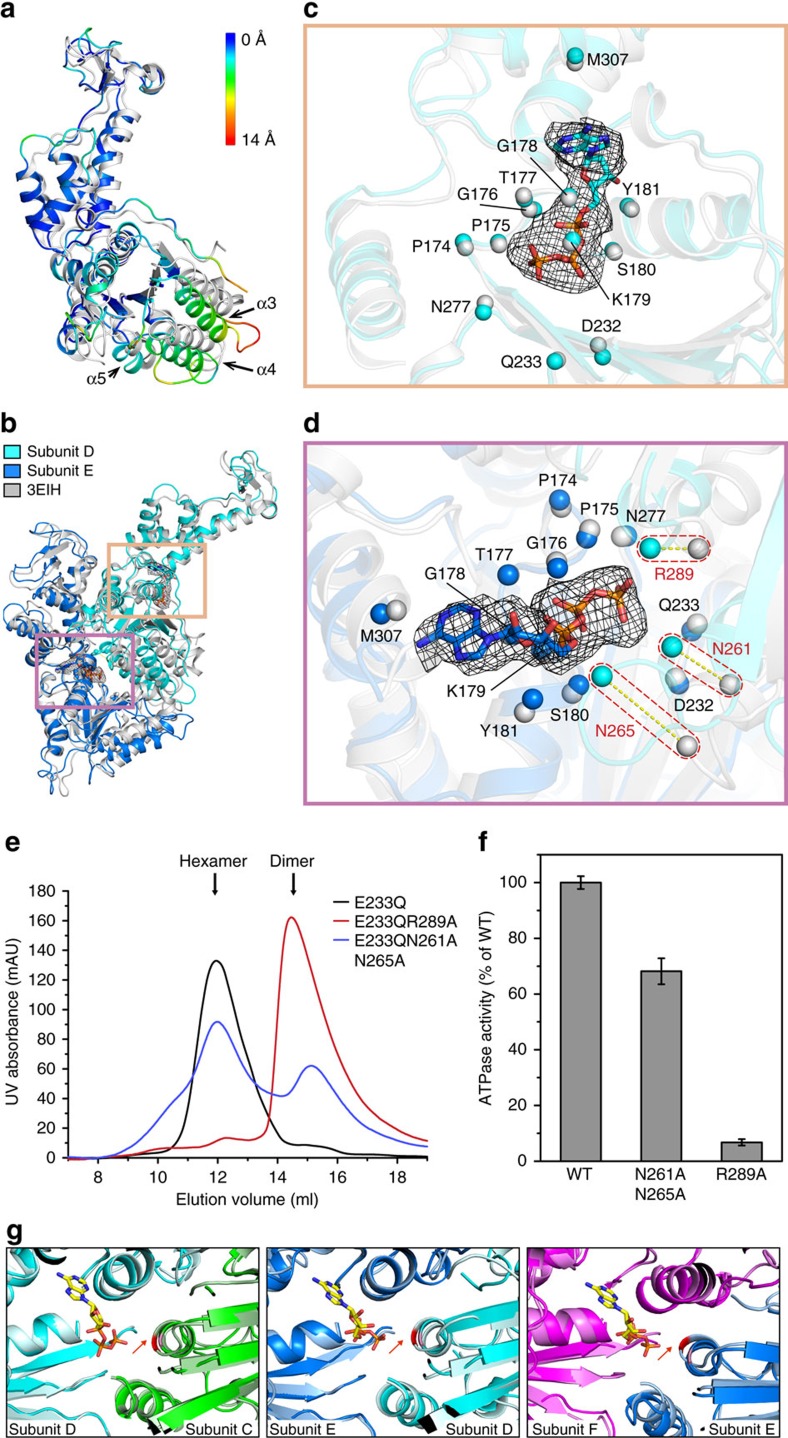
ATPase active centres. (**a**) Comparison of subunit D with the crystal structure of the ATPγS-bound Vps4^E233Q^ (PDB ID: 3EIH)^12^. The crystal structure is coloured grey, and the subunit D is coloured based on the distances between aligned Cα atom pairs with colouring scheme showing on the right. Helixes α3, α4 and α5 are labelled and marked by black arrows. (**b**) A focused view of the adjacent Vps4^E233Q^ subunits D and E with each subunit superimposed with the crystal structure of the ATPγS-bound Vps4^E233Q^. Two ATPase active centres are boxed and detailed in (**c**,**d**). ATP molecules are shown in stick representation, and the map density shown in mesh. (**c**,**d**) Close-up views of the ATPase active centre in subunit D (**c**) and subunit E (**d**). Residues contributing to ATP binding are shown in sphere representation. Large shifts of R289, N261 and N265 in subunit D compared with those in the crystal structure are shown by red dashed boxes in (**d**). The orientations of all close-up views are optimized to show the positions of the residues contributing to ATP binding. All alignments were done using the residues in Walker A loop (Phe174-Tyr181), Walker B (Asp232-Gln233), sensor 1 (Asn277) and M307 as a reference. (**e**) SEC analyses of the oligomeric states of the Vps4 mutants. (**f**) The ATPase activity assays. Data are represented as the average of three independent experiments. Error bars represent s.d. (**g**) Comparison of the ATPase centres of Vta1-free and Vta1-bound Vps4^E233Q^ hexamers. The colouring scheme is the same as in Fig. 1c,e. The ATP molecules in the Vta1-bound Vps4^E233Q^ hexamers are displayed in stick model. The R289 residues (red arrows) in Vta1-free and Vta1-bound Vps4^E233Q^ hexamers are coloured by pink and red, respectively. All alignments were performed using the subunits that the arginine fingers act on *in trans*.

In our structure, we can clearly visualize ATP at the nucleotide-binding pocket of Vps4^E233Q^ protein (Supplementary Fig. 4). This ATP-bound structure of Vps4^E233Q^ provides us the opportunity to compare with the other nucleotide-bound structures to understand the mechanism of the ATP-dependent oligomerization of Vps4. In comparison with the ATPγS-bound Vps4^E233Q^ (PDB ID: 3EIH)^12^, the ATP-bound Vps4^E233Q^ in the hexamer (taking subunit D as representative) has conserved regions contributing to ATP binding including Walker A loop (Phe174-Tyr181), the two negatively charged residues in Walker B (Asp232, and Glu233 that is mutated to Gln233 in our structure), sensor 1 (Asn277) and M307 that can be well aligned with a r.m.s.d. value of 0.884 Å over 12 Cα atoms (Fig. 3a–d). On the other hand, significant differences are observed in the position of the large ATPase domain, especially the surface-exposed helixes including helixes α3, α4 and α5 that are far away from the ATP-binding pocket of the subunit itself, exhibiting a significant movement between these two structures (Fig. 3a,b). Consequently, this change does not affect the conformation of the ATP-binding pocket of the subunit D (Fig. 3c), but instead leads to a relatively compact ATP centre of the neighbouring subunit E by positioning Arg289, Asn261 and Asn265 residues into close proximity to the ATP of subunit E (Fig. 3d), enabling comprehensive close contacts with the ATP to not only allow efficient assembly of the hexamer but also result in elevated ATPase activity. Indeed, mutation of Arg289 to alanine or mutation of both Asn261 and Asn265 to alanines led to reduced ATP-dependent oligomerization activity and the ATPase activity (Fig. 3e,f). Specially, the mutation of Arg289, the highly conserved arginine finger, nearly failed assembly into the hexamer and retained only 7% of the wild-type ATPase activity (Fig. 3e,f). This observation is in agreement with the previous studies of the functions of the arginine finger. The arginine finger residues have been studied extensively and implicated in ATP hydrolysis and oligomer assembly^27^,^28^. As for the Vps4 protein, mutations of the arginine fingers of mouse Vps4B/SKD1 (mmVps4) (R290R291)^13^ and Vps4 from *Sulfolobus solfataricus* (SsoVps4) (R262R263)^21^ impair hexamerization, and arginine finger mutations of SsoVps4^21^ and Vps4 from *M. sedula* (MsVps4) (R259R260)^22^ abolish ATPase activity.

Since Vta1 can increase the ATPase activity of Vps4 *in vitro*, we next examined whether there is any difference in the conformation of the ATPase centre between Vta1-free and Vta1-bound Vps4^E233Q^ hexamers. Comparisons of the ATPase active centres of these two hexamers indicate that in three ATP pockets located at the interface between subunits C and D, D and E, and E and F, the R289 residues of the Vta1-bound Vps4^E233Q^ hexamer clearly shift slightly towards the γ-phosphate of ATP compared with those of the Vta1-free Vps4^E233Q^ hexamer (Fig. 3g). As studied above, the arginine finger R289 is essential for the hexamer assembly and the ATP hydrolysis. In the AAA+ proteins, the arginine finger promotes oligomerization and hydrolysis by interaction with the γ-phosphate of ATP^27^,^28^. Thus, the relatively short distance between the R289 and the γ-phosphate of ATP in the Vta1-bound Vps4^E233Q^ hexamer may facilitate their interactions to help hexamerization and accelerate the ATP hydrolysis.

### Single-molecule assay of ESCRT-III filament disassembly

**Figure 4:**
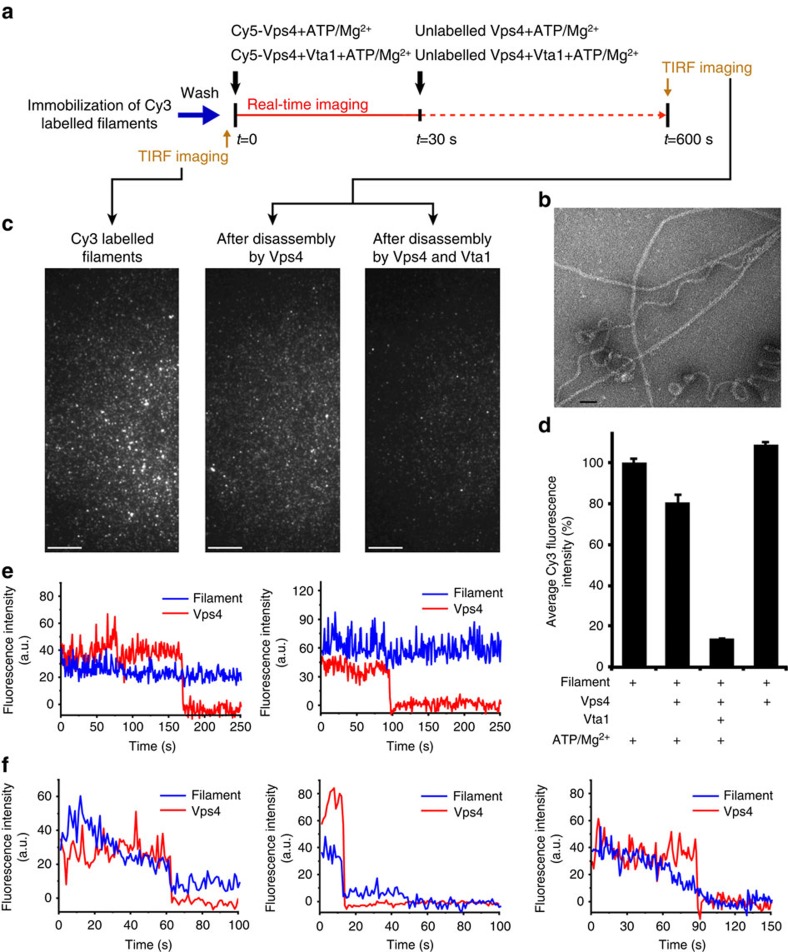
Single-molecule fluorescence assay of the ESCRT-III filament disassembly by Vps4 alone or by Vps4 together with Vta1. (**a**) Procedure of the single-molecule total internal reflection fluorescence (TIRF) microscope experiment. (**b**) Negative staining EM micrograph of the ESCRT-III filaments. Scale bar, 50 nm. (**c**) TIRF microscopy images of Cy3 fluorescence channel representing ESCRT-III filaments after immobilization (left), after disassembly by Vps4 (middle) and after disassembly by Vps4 and Vta1 (right). Scale bar, 10 μm. (**d**) The average Cy3 fluorescence intensities associated with filaments under various conditions of the disassembly reaction. Data are represented as the average of three independent experiments. Error bars represent s.e.m. (**e**,**f**) Single-molecule fluorescence trajectories of Cy5-Vps4 and Cy3-filament during the filament disassembly. (**e**) Cy5-Vps4 disappeared while almost no changes were observed in the filament coupled Cy3 fluorescence intensity. (**f**) Cy5-Vps4 disappeared while the filament coupled Cy3 fluorescence intensity was also decreased.

To further explore how Vta1 affectsVps4 function, we investigated the process of the ESCRT-III filament disassembly by Vps4 alone or by Vps4 together with Vta1 using single-molecule approaches. The experiment procedure is shown in Fig. 4a. We first immobilized Cy3-labelled ESCRT-III filaments (Fig. 4b) composed of the Snf7^R52E^, Vps24 and Vps2 proteins on microscope slides. Snf7^R52E^ is a previously identified Snf7 mutant that is autoactivated and can induce filament formation^5^. Depending on their individual size, each of the ESCRT-III filaments were heavily labelled by tens of or even hundreds of Cy3 fluorophores, and this was confirmed by the fact that fluorescence intensities of filaments were an order of magnitude stronger than single Cy3. Therefore, decreasing of Cy3 signals represented filament disassembly. After washing out unbound filaments, Cy5-labelled Vps4 alone or together with Vta1 was injected, incubated for 30 s and replaced by unlabelled Vps4 alone or together with Vta1. Movies were recorded immediately after labelled Vps4 injection and continued for a total time of 10 min (Fig. 4a). Alternating laser excitation^29^ was used to collect fluorescence intensities of both Cy3 and Cy5 during the process. Average fluorescence intensities of individual Cy3 spots associated with filaments before and after incubating with Vps4 were used to quantitatively represent the degree of filament disassembly (Fig. 4c,d). We observed dramatic decreases in Cy3 fluorescence intensity after 10 min of incubation with Vps4 (Fig. 4c, left and middle, and Fig. 4d). As a control, no significant decrease was observed in the absence of ATP (Fig. 4d). Thus, the decreases of Cy3 fluorescence intensity depended on the ATP hydrolysis by Vps4, indicating that the disassembly of ESCRT-III filaments by Vps4 was reconstituted on our single-molecule fluorescence assay. In addition, we captured more dramatic decreases of Cy3 fluorescence intensity when Vta1 was present with Vps4 (Fig. 4c, right, and Fig. 4d) and significant increases of filament disassembly rates in the presence of Vta1 (Supplementary Table 2), indicating that Vta1 promotes the Vps4 ability of disassembling the ESCRT-III filaments. We next selected the spots containing both Cy5-Vps4 and Cy3-filament and carefully examined the evolution of their fluorescence intensities during the 10 min disassembly process. In many cases, Cy5-Vps4 disappeared while almost no changes were observed in the filament coupled fluorescence intensity (Fig. 4e), indicating the filament was not disassembled. Thus, these associations represent nonproductive binding events. In the remaining cases, Cy5-Vps4 disappeared while the filament coupled Cy3 fluorescence intensity also decreased (Fig. 4f). Interestingly, in these cases we typically observed that fluorescence intensities of the Cy5-Vps4 and the Cy3-filament disappeared simultaneously (Fig. 4f), although a few cases displayed the gradual decreasing of Cy3 before Cy5-Vps4 disappearance. Clearly, these single-molecule events represented Vps4 molecules actively disassembling the filaments. The dwell times of Cy5-Vps4 in the absence and presence of Vta1 before their disappearances were 139±12 and 90±7 s, respectively, indicating that Vps4 stably associated with the filament for at least 90 s during the process of the filament disassembly. Otherwise, transient bound Cy5-Vps4 on the filaments would be quickly replaced by 100 nM unlabelled Vps4 presence in the solution. Furthermore, we found that the percentage of the active disassembly events significantly increases from 4.5% (*n*=243) for Vps4 alone to 19.2% (*n*=276) when additional Vta1 was present, suggesting more active Vps4 recruited to the filaments in the presence of Vta1. Since both our structures of the ATP-bound Vps4 and its complex with Vta1 and the previous studies of the oligomeric state of the Vps4 (ref. 21) indicate that the active form of Vps4 is hexameric, this observation in the single-molecule filament disassembly assays further suggests that more Vps4 hexamers recruited to the filaments in the presence of Vta1; that is, Vta1 serves as an assembly factor to help Vps4 oligomerize into active hexamers associated with filaments.

In this study, we present the cryo-EM structure of the ATP-bound Vps4^E233Q^ hexamer that clearly demonstrates that the higher-order oligomer formed by Vps4^E233Q^ upon binding to ATP is a hexamer. Previous studies reported that Vps4^E233Q^ mutant assembled into higher-order oligomer containing 8–12 subunits in the presence of ATP by the gel filtration analysis^6^,^8^,^12^,^21^,^25^. However, molecular mass determination by gel filtration may be not accurate due to the poor resolution of the gel filtration retention times at this high-molecular-weight range and the effects of the molecule shapes on the measurement. Two EM structural studies also showed that Vps4^E233Q^ mutant assembled into double-ring structures with 12 subunits^24^,^25^, and one EM study using wild-type Vps4 lacking the MIT domain and non-hydrolysable ATP analogues (AMPPNP) reported the Vps4 oligomer with 14 subunits, but these structures are all at a low-resolution range between 18 and 34 Å, and therefore the interpretation of these structures may not be reliable. Although we also used the E233Q mutant for the oligomer assembly, our near-atomic structures clearly reveal the assembled oligomer is a hexamer (Fig. 1), and this is also consistent with our MALS result showing a molecular weight of ∼300 kD of the assembled oligomer (Supplementary Fig. 1a), corresponding to six Vps4 molecules in the oligomer.

In most published Vps4 crystal forms, Vps4 subunits assembled into a continuous helix with a sixfold screw axis, and if viewed along the screw axis, the projection of these molecules forms a hexameric ring^8^,^11^,^12^,^13^,^20^,^21^,^22^. It should be noted that the arrangement of the Vps4 subunits in the crystal forms is different from that in our ATP-bound Vps4^E233Q^ hexamer. The former is an opened helical assembly^8^,^11^,^12^,^13^,^20^,^21^,^22^, while the latter is a closed spiral-shaped ring-like arrangement (Fig. 1b–g). Accordingly, the interface of the adjacent Vps4 subunits is also different. The helix in the crystal forms is stabilized by extensive interactions between the large and small AAA ATPase domains of the adjacent Vps4 subunits^12^. These interactions are well preserved in our ATP-bound Vps4^E233Q^ hexamer (Fig. 2b,d,g). Moreover, our structure discovered interactions between the large ATPase domains and between the C-terminal helices essential for the hexamer assembly (Fig. 1b,c,e,f,h). Contrasting the large and small ATPase domain interactions, which are predominated by van der Waals contacts (Fig. 2d,g), the large ATPase domain interactions comprise mostly polar contacts (Fig. 2c,f). Compared with hydrophobic interactions, polar interactions usually exhibit faster kinetics and thus are more amenable to the biological function—in this case, ESCRTIII filament disassembly. Additionally, the interface between adjacent large ATPase domains is close to the centre pore of the hexameric ring that is essential for the function of Vps4. Therefore, the atomic interactions at this interface appear not only to be important for hexamer assembly, but also to have evolved to facilitate the underlying biological function.

One finding revealed by our structures is the split spiral arrangement of Vps4^E233Q^ subunits in both the Vps4^E233Q^ hexamer (Fig. 1f,g) and its complex with Vta1 (Supplementary Fig. 6c), and the biological relevance of the subunit interface in the hexamer was confirmed by the ATPase activity assays of the site-directed mutant proteins (Supplementary Fig. 8). The structural observation is reminiscent of that in other two AAA+ ATPase members: the transcriptional activator NtrC1 (nitrogen regulatory protein C1) and the NSF (*N*-ethylmaleimide-sensitive factor)^30^,^31^. Besides these AAA+ ATPase members, the split spiral arrangement has been observed for some helicases, including the E1 helicase of bovine papiloma virus^32^, the DnaB helicase from *Geobacillus stearothermophilus*^33^ and the eukaryotic MCM (minichromosome maintenance protein complex) helicases^34^,^35^. These ATPases have very different biological functions: NtrC1 is suggested to remodel σ54 protein by directly interacting with region I that is predicted to form an α-helix^30^,^36^,^37^; NSF disassembles SNARE complex that is a four-helix bundle^38^,^39^,^40^; helicases unwind DNA that has a helical nature. Thus, the unifying structural feature of the split spiral-like ring likely plays a key role for these ATPases to exert forces derived from ATP hydrolysis on their target macromolecules.

## Methods

### Protein expression and purification

*Saccharomyces cerevisiae* Vps4, Vta1, Snf7, Vps24 and Vps2 genes were amplified by PCR from yeast genomic DNA and directionally cloned into the pET28a vector (Novagen) with an N-terminal His_6_ tag. Constructs expressing mutant proteins were generated with Quick Change Mutation Kit (Transgen) and verified by DNA sequencing. All proteins were expressed in *Escherichia coli* BL21 (DE3) strains and induced with 0.5 mM isopropyl-β-D-thiogalactopyranoside at 21 °C overnight. The cells were lysed by sonication suspended in the lysis buffer containing 20 mM HEPES at pH 7.5, 500 mM NaCl, 10 mM imidazole, 1 mm phenylmethylsulfonyl fluoride and 2 mM β-mercaptoethanol (β-ME). The clarified lysates were purified on a Ni–nitrilotriacetic acid column (GE Healthcare). The eluates were incubated with 2 U ml^−1^ apyrase (Sigma-Aldrich) at 4 °C overnight. All proteins were further purified by SEC using a Superdex 200 10/300 GL column (GE Healthcare) in the fast performance liquid chromatography (FPLC) buffer containing 20 mM HEPES at pH 7.5, 100 mM NaCl and 1 mM dithiothreitol. The purity and quality of all the proteins were tested by SDS–PAGE.

### Vps4^E233Q^ hexamer and Vps4^E233Q^–Vtal complex assembly for EM

Assembly of Vps4 oligomer was completed through incubation of the purified E233Q protein with 1 mM ATP at 4 °C for 1 h. The mixture was then subjected to a Superdex 200 10/300GL column (GE Healthcare) in the assembly buffer containing 20 mM HEPES at pH 7.5, 100 mM NaCl, 1 mM dithiothreitol, 2 mM MgCl_2_ and 1 mM ATP. For the reconstitution of the complex between Vps4^E233Q^ hexamer and Vta1, E233Q protein was mixed with a 1.5- to 2-fold molar excess of Vta1 and then incubated with 1 mM ATP at 4 °C for 1 h. The mixture was then subjected to a Superose 6 10/300GL column (GE Healthcare) in the assembly buffer.

### SEC with MALS

SEC–MALS experiments were performed using an Agilent 1200 high-performance liquid chromatography system (Agilent Technologies, Santa Clara, CA, USA) coupled to a Wyatt DAWN HELEOS-II MALS instrument and a Wyatt Optilab rEX differential refractometer (Wyatt, Santa Barbara, CA, USA). For chromatographic separation, 200 μl of 5 mg ml^−1^ (50 μM) Vps4^E233Q^ protein preincubated with 1 mM ATP at 4 °C for 1 h was injected onto a Superdex 200 10/300 GL column (GE Healthcare) at a flow rate of 0.3 ml min^−1^ in the assembly buffer. The outputs were analysed by the ASTRA 6.0 software (Wyatt). MALS signals, combined with the protein concentration determined by the refractive index, were used to calculate the molecular mass.

### SEC analysis of oligomeric states and protein interactions

To examine the oligomeric states of Vps4 mutants, each mutant protein at 100 μM was incubated with 1 mM ATP at 4 °C for 1 h, and then loaded onto a Superdex 200 10/300 GL column (GE Healthcare) preequilibrated with the assembly buffer. To examine the interactions between Vps4 and Vta1 in the absence of ATP, Vps4 mutants at 100 μM were incubated with Vta1 at a molar ratio of 1:1.5 in the absence of ATP at 4 °C for 1 h before being loaded onto the Superdex 200 10/300 GL column (GE Healthcare) preequilibrated with the FPLC buffer. To examine the interactions between Vps4 and Vta1 in the presence of ATP, Vps4 mutants at 5 μM were incubated with Vta1 at a molar ratio of 1:2 in the presence of 1 mM ATP at 4 °C for 1 hour before being loaded onto the Superose 6 10/300 GL column (GE Healthcare) preequilibrated with the assembly buffer.

### Stability assay

The formed Vta1-free Vps4^E233Q^ hexamers and the Vta1-bound Vps4^E233Q^ hexamers eluted from SEC in the assembly buffer were individually reloaded onto the Superose 6 10/300 GL column (GE Healthcare) in the FPLC buffer. For the apyrase treatment, the 10 U ml^−1^ apyrase (Sigma-Aldrich) was added to the samples and incubated at 4 °C overnight before SEC.

### ATPase activity assay

ATPase activity assays measure released phosphate based on the formation of coloured complex between malachite green reagent and free phosphate according to the manufacturer’s instructions using the QuantichromeTM ATPase/GTPase Assay Kit (BioAssay Systems). Briefly, 1 μM of wild-type or mutant Vps4 proteins in the absence or presence of Vta1 in the ATPase assay buffer containing 20 mM HEPES at pH 7.5, 100 mM NaCl, 2 mM MgCl_2_ and 5 mM ATP were incubated at 37 °C for various times (0–4 min), and stopped by adding reagent from the kit. The solution was incubated at room temperature for 30 min, and immediately the absorbance at 620 nm was detected using a plate reader (Enspire, Perkin Elmer). The released phosphate was calculated based on the absorbance standard curve established using KH_2_PO_4_ standards.

### ATP/ADP quantification assay

The 50 μM Vps4^E233Q^ and 100 μM Vta1 protein were incubated for 1 h on ice in the assembly buffer, and then loaded onto the Superose 6 10/300GL column (GE Healthcare) in the FPLC buffer. The eluted Vps4^E233Q^–Vta1 complex was used to examine the ATP and ADP levels by the bioluminescent detection-based method (Bioassay System EnzyLight ADP Assay Kit, EADP-100) according to the manufacturer’s instructions. The luminescence values were detected using a plate reader (Enspire Multimode Plate Reader, Perkin Elmer). The nucleotide levels were calculated based on the luminescence standard curves established using ATP and ADP standards. Three independent experiments were carried out on at least two different batches of purified Vps4^E233Q^ and Vta1 proteins and each experiment was carried out in at least triplicate.

### Stoichiometry determination

We used the fluorescence assay-based method to evaluate the stoichiometry of the ATP-bound Vps4^E233Q^–Vta1 complex. We constructed the single-cysteine mutants (containing only one cysteine residue) of Vps4 (Vps4^E233QC376S^) and Vta1 (Vta1^C124S154S^) through site-directed mutagenesis and purified them as for other mutant proteins. The concentrations of all proteins were measured by Pierce BCA Protein Assay Kit (Thermo). Vps4 and Vta1 proteins were concentrated to 4 and 10 mg ml^−1^ in the labelling buffer containing 50 mM HEPES, 100 mM NaCl and 1 mM TCEP (tris(2-carboxyethyl)phosphine), pH 7.4, respectively. Cy5 maleimide and Cy3 maleimide (GE Healthcare) were added into the Vps4 and Vta1 protein solutions, respectively, at the protein/fluorophore ratio of 1:4. Labelling reactions were performed on ice overnight, and terminated by 3 mM β-ME. The free dyes were removed by using the NAP-5 columns. Then, we generated the ATP-bound Vps4^E233Q^–Vta1 complex using these labelled proteins by incubating them at the molar ratio of 1:2 in the presence of ATP on ice for 1 h and then applying the mixtures to SEC in the assembly buffer to separate the complex. The fluorescence intensities of Cy5 and Cy3 of the complex were measured using a plate reader (Enspire Multimode Plate Reader, Perkin Elmer). Then, the protein concentrations of Vps4 and Vta1 in the complex were calculated according to the fluorescence intensities and the standard curves of the protein concentration versus the fluorescence intensity. Three independent experiments were carried out and each experiment was carried out in at least triplicate.

### Cryo-EM sample preparation and data collection

The assembled ATP-bound Vps4^E233Q^ hexamer eluted from SEC was concentrated to ∼1 mg ml^−1^, and incubated with 0.02% glutaraldehyde for ∼5 min on ice. Then, aliquots of 4 μl of this sample were applied to the glow-discharged Quantifoil R1.2/1.3 holey carbon grids (Quantifoil Micro Tools GmbH), blotted for 2 s and plunge-frozen by using FEI Vitrobot Mark IV. The sample of the ATP-bound Vps4^E233Q^–Vta1 complex was prepared same as the Vps4^E233Q^ hexamer, except without glutaraldehyde treatment. Grids were examined using an FEI Titan Krios operated at 300 kV, and images were recorded using a K2 Summit direct electron detector (Gatan) in superresolution mode, at a nominal magnification of 22,500, and with the defocus ranging from −1.5 to −3.0 μm. Images were collected under low-dose condition in a semiautomatic manner using UCSF-Image4 (ref. 41). The dose rate on the camera was set to be 8.2 electrons per pixel per second. For each micrograph stack, a total of 32 frames were collected with an exposure time of 8 s, leading to a total accumulated dose of 50 electrons per Å^2^ on the specimen.

### Image processing

The collected original micrograph stacks were aligned and summed using whole-image motion correction^41^, and binned twofold, resulting in a pixel size of 1.30654 Å per pixel. The defocus values of micrographs were estimated by CTFFIND3 (ref. 42). The particles were picked by EMAN2 (ref. 43) and RELION 1.3 (refs 44, 45). All 2D and 3D classifications and refinements were performed using RELION 1.3 (refs 44, 45).

A total of 31,883 and 66,905 particles were first boxed from 100 micrographs of the ATP-bound Vps4^E233Q^ hexamer and 120 micrographs of the ATP-bound Vps4^E233Q^–Vta1 complex using e2boxer.py in EMAN2 (ref. 43), respectively. Then, the boxed particles were extracted and reference-free 2D class averaging was performed. The generated 2D class averages were used as the templates for the subsequent autopicking of particles using RELION 1.3 (refs 44, 45). A total of 467,563 particles of the ATP-bound Vps4^E233Q^ hexamer and 1,644,872 particles of the ATP-bound Vps4^E233Q^–Vta1 complex were automatically picked out from 1,042 and 1,263 micrographs, respectively, and 2D classified using RELION 1.3 (refs 44, 45). Then 411,144 Vps4^E233Q^ particles and 157,109 Vps4^E233Q^–Vta1 particles were empirically selected from good classes of 2D classifications. We first performed 3D auto-refine using all 411,144 Vps4^E233Q^ particles and all 157,109 Vps4^E233Q^–Vta1 particles with a disk-shaped volume generated from EMAN2 (ref. 43) used as an initial model, respectively. The obtained reconstructions low-pass filtered to 60 Å were then used as the references of the 3D classifications. All of the 411,144 Vps4^E233Q^ particles were divided into eight classes. The most homogeneous class of 106,918 particles was subjected to auto-refinement without any symmetry imposed and resulted in a reconstruction at an overall resolution of 4.1 and 3.9 Å after applying a soft mask around the rigid part (excluding the subunit A) based on the gold-standard Fourier shell correlation (FSC) 0.143 criterion^46^. All of the 157,109 Vps4^E233Q^–Vta1 particles were divided into six classes. Auto-refinement of the best class of the 106,106 particles without any symmetry imposed resulted in a reconstruction of 4.3 and 4.2 Å after applying a soft mask around the rigid part (excluding the subunit A and all bound Vta1 proteins). The handedness of the maps were determined and corrected by comparison with crystal structure of the ATPγS-bound Vps4^E233Q^ (PDB ID: 3EIH)^12^. Additional cycles of 3D classifications and refinements did not further improve the overall resolution of the maps. Local resolution was estimated using ResMap^47^.

### Model building

For building the model of the ATP-bound Vps4^E233Q^ hexamer, the crystal structure of the ATPγS-bound Vps4^E233Q^ (PDB ID: 3EIH, chain A)^12^ was docked into the density map using Chimera^48^. The structure was manually adjusted based on the density map in Coot^49^. Next, the model was refined by Phenix^50^ in real space with stereochemical and secondary structure restraints, and further refined by REFMAC in Fourier space^51^,^52^. The model of the ATP-bound Vps4^E233Q^–Vta1 complex was built in the same way as the model of the ATP-bound Vps4^E233Q^ hexamer using the available crystal structures of the ATPγS-bound Vps4^E233Q^ (PDB ID: 3EIH)^12^ and the Vta1 CTD dimers (PDB ID: 2RKL)^19^ as the starting models to refine. The atomic models were cross-validated according to previously described procedures^53^. Briefly, the coordinates of the final model were randomly displaced by 0.5 Å using the Phenix (PDB Tools) to remove potential model bias. The displaced model was then refined against one of the half maps (produced from a half set of all particles during refinement by RELION). FSC curves were calculated between the resulting model and half1 map (model versus half1 map, FSC_work_, that is, used for refinement), the resulting model and half2 map (model versus half2 map, FSC_free_, that is, not used for refinement) and the resulting model and the final density map (model versus summer map) from all particles. The lack of significant separation between work and free FSC curves suggested that the models were not overfitted.

MolProbity^54^ (http://molprobity.biochem.duke.edu/) and EMRinger^55^ were used to evaluate the final models, and final statistics of the model was provided in the Supplementary Table 1. Interface areas were calculated by PISA^56^. Pymol (http://www.pymol.org/) and Chimera^48^ were used for structural analysis and figure preparation.

### ESCRT-III filament reconstitution

The ESCRT-III filament was reconstituted by incubating Snf7^R52E^, Vps24 and Vps2 in the concentrations of 50, 25 and 25 μM, respectively, at 30 °C for 30 min (ref. 5). Then, the formed filaments were centrifuged at 50,000 r.p.m. for 30 min in a TLA-100 ultracentrifuge rotor (Beckman Coulter). Pellet was resuspended in the buffer containing 50 mM HEPES at pH 7.4, 100 mM NaCl and 1 mM TCEP. All the filaments were verified by SDS–PAGE and negative staining.

### ESCRT-III filament disassembly assay

The reconstituted ESCRT-III filaments were incubated with 1 μM Vps4 in the buffer containing 20 mM HEPES at pH 7.4, 2 mM ATP, 2 mM MgCl_2_, 1 mM TCEP at 25 °C for 5 min (Supplementary Fig. 10a) or indicated time points (Supplementary Fig. 10b) and then 50 mM EDTA were added to stop the reactions. The samples were centrifuged at 50,000 r.p.m. for 30 min in a TLA-100 rotor, and the supernatant and pellet were analysed by SDS–PAGE. The intensities of bands of Snf7 were quantified by ImageJ. Each experiment was carried out independently for three times.

### Vps4 protein and ESCRT-III filament labelling

Vps4 proteins were concentrated to 2 mg ml^−1^ in labelling buffer containing 50 mM HEPES, 100 mM NaCl and 1 mM TCEP, pH 7.4. Cy5 maleimide (GE Healthcare) was added into the protein solution at protein/fluorophore ratio of 1:4. Labelling reactions were performed on ice overnight, and terminated by 3 mM β-ME. The free dyes were removed by using a NAP-5 column. The labelling efficiency was 48%.

The reconstituted filaments were used for labelling with the Cy3 dye and biotin. Filaments, Cyanine3 NHS (Lumiprobe) and NHS-biotin (Thermo Scientific) were incubated at a molar ratio of 10:10:1 (Snf7^R52E^ monomer:Cyanine3 NHS:NHS-biotin) in the labelling buffer on ice for 2 h. The free dyes and biotins were removed by centrifugation at 50,000 r.p.m. for 30 min. The labelled filaments were precipitated and resuspended in 50 mM HEPES at pH 7.4, 100 mM NaCl. Because NHS esters (*N*-hydroxysuccinimide esters) target and crosslink with primary amines on the proteins and reconstituted filaments are formed by tens of or hundreds of monomers, each filament was expected to be labelled with tens of Cyanine3 and several biotin. Although it is hard to estimate the labelling efficiency of filaments, they can be specifically attached to our coverslip via streptavidin coating and exhibit an order of magnitude stronger fluorescence intensity than single Cyanine3 fluorophore. Therefore, in our single-molecule assay, decreasing of Cyanine3 (Cy3) fluorescence signals was used to quantify dissembling of the reconstituted filaments.

Bulk experiments and single-molecule experiments demonstrated that Cy5-labelled Vps4 exhibits similar filament disassembly activities as unlabelled Vps4 and Cy3/biotin-labelled filaments could be disassembled with similar efficiency as unlabelled filaments by Vps4 (Supplementary Fig. 10 and Supplementary Table 2).

### Acquisition and analysis of single-molecule data

Filaments were specifically attached to microscope flow cells formed by polyethylene glycol-passivated slides, decorated with biotin-polyethylene glycol and streptavidin^57^. Single-molecule experiments were performed at 23 °C in the buffer containing 50 mM HEPES at pH 7.4, 100 mM NaCl with an oxygen scavenging system, containing 3 mg ml^−1^ glucose, 100 μg ml^−1^ glucose oxidase (Sigma-Aldrich), 40 μg ml^−1^ catalase (Roche), 1 mM cyclooctatetraene (Sigma-Aldrich), 1 mM 4-nitrobenzylalcohol (Sigma-Aldrich), 1.5 mM 6-hydroxy-2,5,7,8-tetramethyl-chromane-2-carboxylic acid (Trolox, Sigma-Aldrich) added from a concentrated DMSO stock solution and 1% bovine serum albumin (Amresco) to reduce nonspecific binding. After filament immobilization, 100 nM of Vps4 labelled with Cy5 alone or together with 500 nM Vta1 was injected with 1 mM ATP and 1 mM Mg^2+^. After incubation for 30 s, 100 nM unlabelled Vps4 alone or together with 500 nM Vta1 was injected along with 1 mM ATP and 1 mM Mg^2+^ to wash out labelled Vps4 in the microscope flow cell, and further incubated for a total time of 10 min. Movies were recorded immediately after labelled Vps4 injection and continued for 10 min. Fluorescence images of filaments were taken before Vps4 injection and after Vps4 incubation (Fig. 4a). Under our experimental conditions, rate of Cy5 photobleaching was 0.00071±0.00005, s^−1^ (average±s.e.m.)

Single-molecule spectroscopic microscopy was performed on a home-built objective-type total internal reflection fluorescence microscope, based on a Nikon Eclipse Ti-E with an EMCCD camera (Andor iXon Ultra 897), and solid-state 488, 532 and 640 nm excitation laser (Coherent Inc., OBIS Smart Lasers). To monitor fluorescence intensities of filaments and Vps4, alternating laser excitation^29^ between two lasers excitation was achieved by synchronizing laser on–off switch with EMCCD frames. Fluorescence emission from the probes was collected by the microscope and spectrally separated by interference dichroic (T635lpxr, Chroma) and bandpass filters, ET585/65m (Chroma, Cy3) and ET700/75m (Chroma, Cy5), in a Dual-View spectral splitter (Photometrics, Inc., Tucson, AZ, USA). All movies were collected using Cell Vision software (Beijing Coolight Technology). Single-molecule events longer than three frames were picked by home-written Matlab scripts.

### Data availability

The reconstructed density maps were deposited to Electron Microscopy Data Bank with accession code EMD-6735 (without mask), EMD-6733 (with mask), EMD-6736 (without mask) and EMD-6734 (with mask), and the atomic coordinates to Protein Data Bank with PDB ID code 5XMI and 5XMK for the ATP-bound Vps4^E233Q^ hexamer and ATP-bound Vps4^E233Q^–Vta1 complex, respectively. The data that support the findings of this study are available from the corresponding authors on request.

## Additional information

**How to cite this article:** Sun, S. *et al*. Cryo-EM structures of the ATP-bound Vps4^E233Q^ hexamer and its complex with Vta1 at near-atomic resolution. *Nat. Commun.* **8,** 16064 doi: 10.1038/ncomms16064 (2017).

**Publisher’s note:** Springer Nature remains neutral with regard to jurisdictional claims in published maps and institutional affiliations.

## Supplementary Material

Supplementary Information
