# Supplementary material for: Cryo-EM structures of the ATP-bound Vps4E233Q hexamer and its complex with Vta1 at near-atomic resolution
Source: Nat Commun. 2017 Jul 17;8:16064. doi: 10.1038/ncomms16064 (PMC5520056; doi:10.1038/ncomms16064)
Supplement: Supplementary Information [file ncomms16064-s1.pdf]

File Name: Supplementary Information
Descriptions: Supplementary Figures and Supplementary Tables

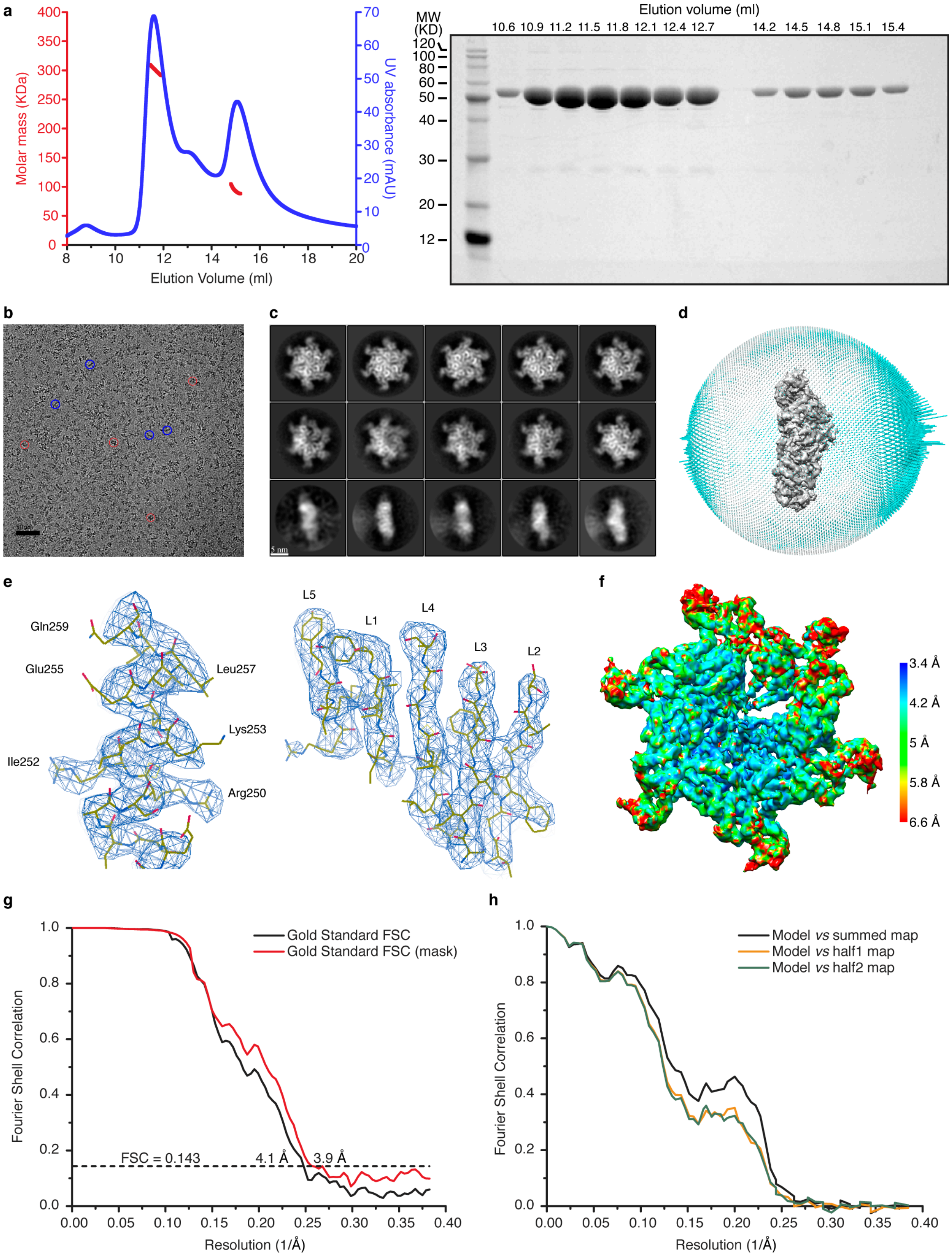

Supplementary Figure 1 Purification and cryo-EM analysis of the ATP-bound Vps4^{E233Q} hexamer.

(a) Multiangle light scattering (MALS) of the ATP-bound Vps4^{E233Q} hexamer (left panel) and the SDS-PAGE gel (12% resolving gel) of the corresponding fractions (right panel).

The molecular weights of the two major peaks were determined as ~300 kDa and ~100 kDa, corresponding to $Vps4^{E233Q}$ hexamer and dimer, respectively. **(b)** A representative electron micrograph of the $Vps4^{E233Q}$ hexamer low pass filtered to 5 Å. A few typical protein particles of top views and side views are marked by red and blue circles, respectively. **(c)** Typical good reference-free 2-D class averages from the single particle images of the $Vps4^{E233Q}$ hexamer. **(d)** Euler angle distribution of particles contributing to the final reconstruction of the $Vps4^{E233Q}$ hexamer. Each cylinder represents one view and the size of the cylinder is proportional to the number of particles for that view. **(e)** Examples of the densities in the cryo-EM map of the $Vps4^{E233Q}$ hexamer, including an α helix (residues 249-260) and the β sheet (residues 168-172 for strand 1, 193-198 for strand 2, 227-232 for strand 3, 270-276 for strand 4, 293-295 for strand 5) from subunit C. The atomic models are displayed in stick representation, and the density maps were showed as blue mesh. Segmented density maps are displayed at ~ 6 σ contour level. **(f)** The EM density map of the $Vps4^{E233Q}$ hexamer color-coded to show the local resolution as estimated by ResMap. **(g)** Gold-standard FSC curves of the reconstruction of the $Vps4^{E233Q}$ hexamer. The reconstruction gives an overall resolution of 4.1 Å, and 3.9 Å after applying a soft mask around the rigid part (excluding the subunit A). **(h)** FSC curves for the cross-validation of the atomic models of the $Vps4^{E233Q}$ hexamer. The lack of significant separation between work and free FSC curves suggested that the models were not overfitted. See Methods for details.

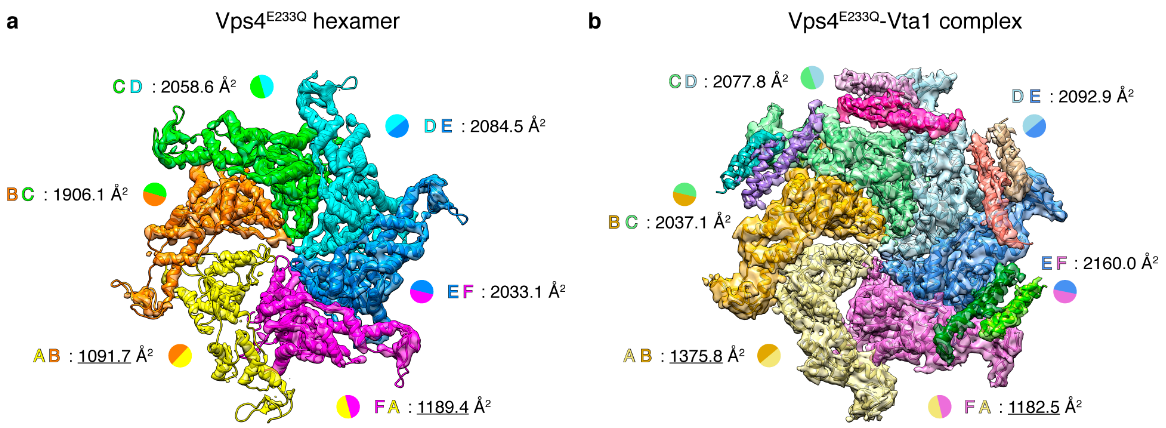

Supplementary Figure 2 Docking of the atomic models into the density maps of the ATP-bound Vps4^{E233Q} hexamer and its complex with Vta1.

Docking of the atomic models into the density maps of the ATP-bound Vps4^{E233Q} hexamer **(a)** and the ATP-bound Vps4^{E233Q}-Vta1 complex **(b)**. The interface areas between Vps4^{E233Q} subunits in the ATP-bound Vps4^{E233Q} hexamer (a) and in the ATP-bound Vps4^{E233Q}-Vta1 complex (b) are also shown. The coloring scheme is the same as in Fig. 1c,e. The interface areas of subunits A and its neighboring subunits (~1000 Å^2 buried surface), which are only about half of those of other pairs of subunits (~2000 Å^2 buried surface), are underlined.

a

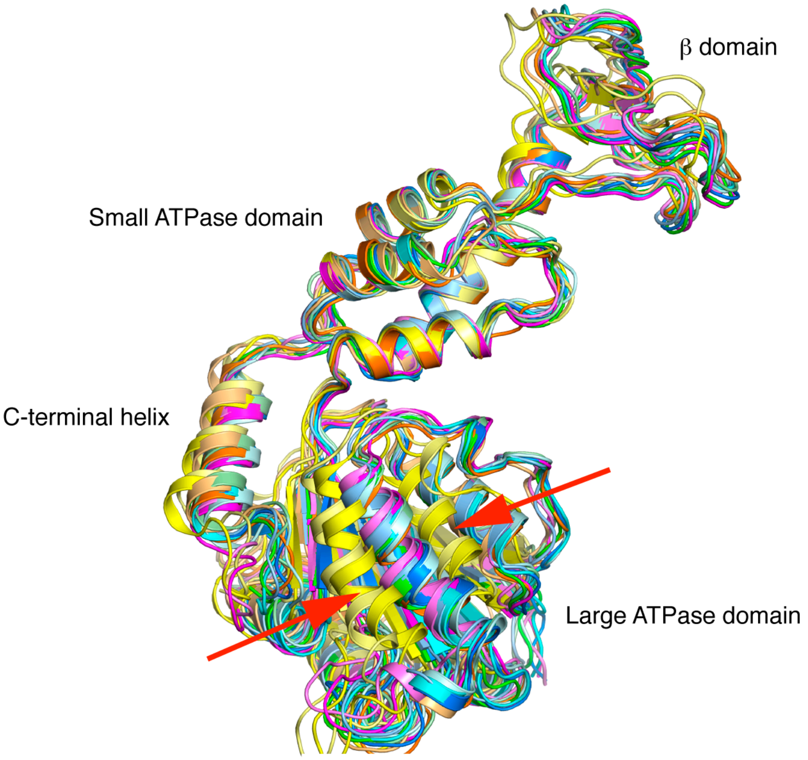

b

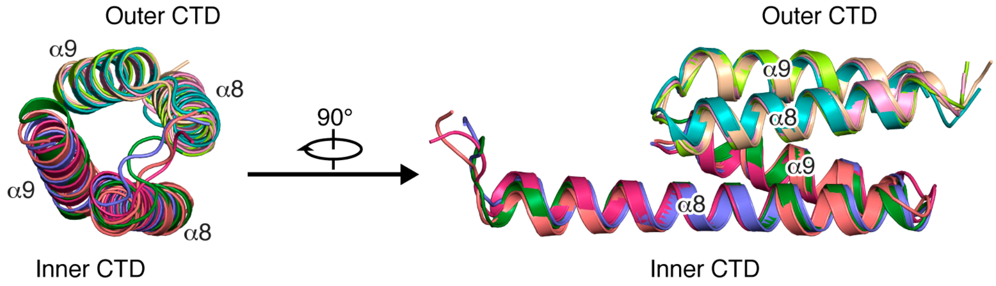

Supplementary Figure 3 Superimposition of Vps4^{E233Q} subunits and Vta1 CTD dimers.

(a) Comparison of the twelve Vps4^{E233Q} subunits from the Vta1-free Vps4^{E233Q} hexamer and the Vta1-bound Vps4^{E233Q} hexamer. The coloring scheme is the same as in Fig. 1c,e. Note that the large ATPase domains of subunits A (red arrows) from both the Vta1-free Vps4^{E233Q} hexamer and the Vta1-bound Vps4^{E233Q} hexamer exhibit different positions relative to the small ATPase domains, compared to other subunits. **(b)** Alignment of the four Vta1 CTD dimers from the ATP-bound Vps4^{E233Q}-Vta1 complex. The coloring scheme is the same as in Fig. 1e. All of the CTD dimers are well aligned.

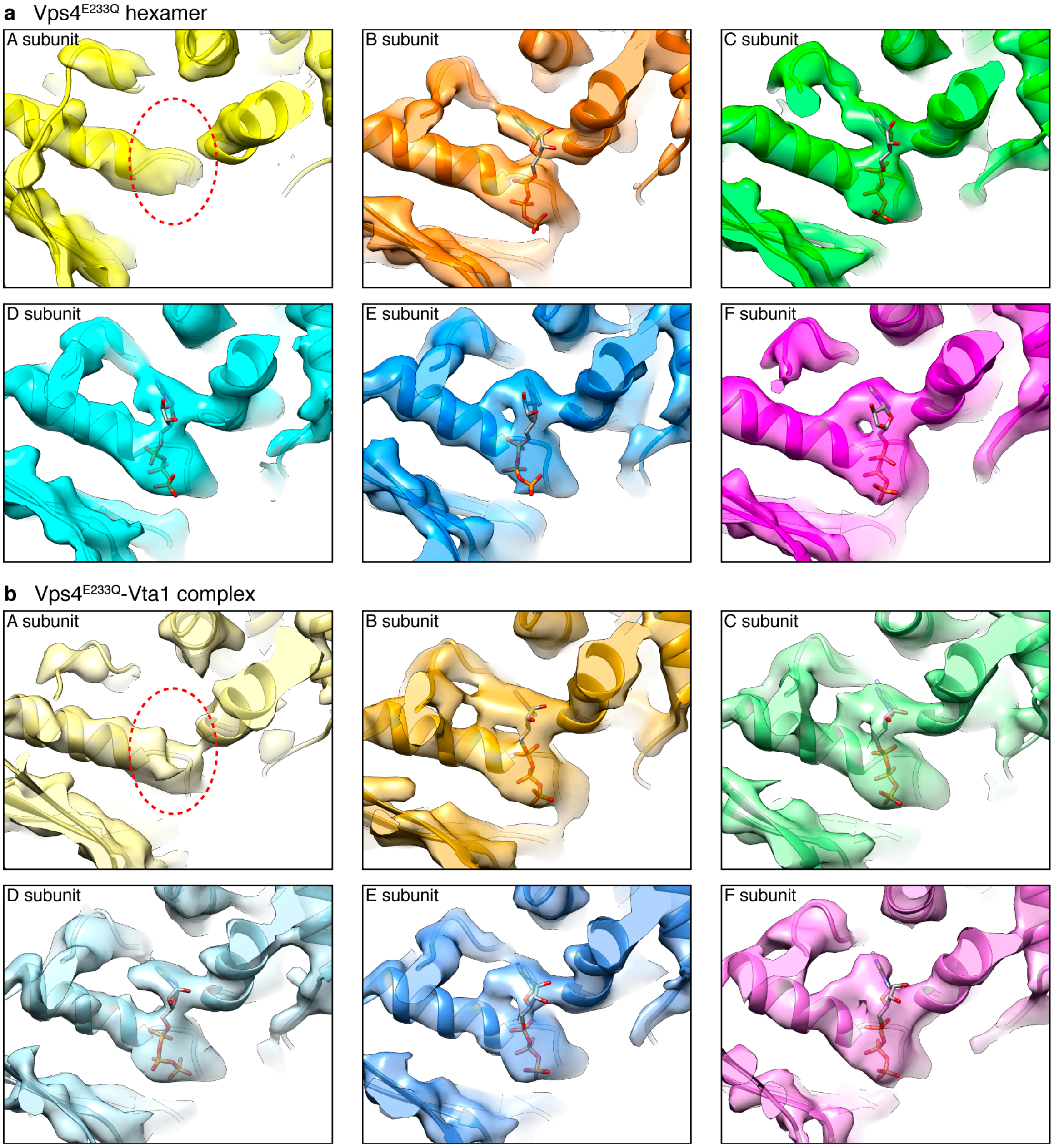

Supplementary Figure 4 Nucleotide binding sites of the Vps4^{E233Q} subunits.

(a) Zoomed-in views of the ATPase active centers for all six Vps4^{E233Q} subunits from the ATP-bound Vps4^{E233Q} hexamer. The density maps are contoured at the 5.8 σ level for subunits B-F, and the 5.0 σ level for subunit A. The coloring scheme is the same as in Fig. 1c. **(b)** Zoomed-in views of the ATPase active centers for all six Vps4^{E233Q} subunits from the ATP-bound Vps4^{E233Q}-Vta1 complex. The density maps are contoured at the 6.8 σ level for subunits B-F, and the 5.6 σ level for subunit A. The coloring scheme is the same as in Fig. 1e. The EM densities are shown together with the built atomic model with

protein in cartoon representation and nucleotide in stick representation. The positions of the nucleotide in subunit A are marked by red dashed circles. Five out of the six Vps4^{E233Q} subunits of both the ATP-bound Vps4^{E233Q} hexamer and the ATP-bound Vps4^{E233Q}-Vta1 complex show clear densities for the nucleotide.

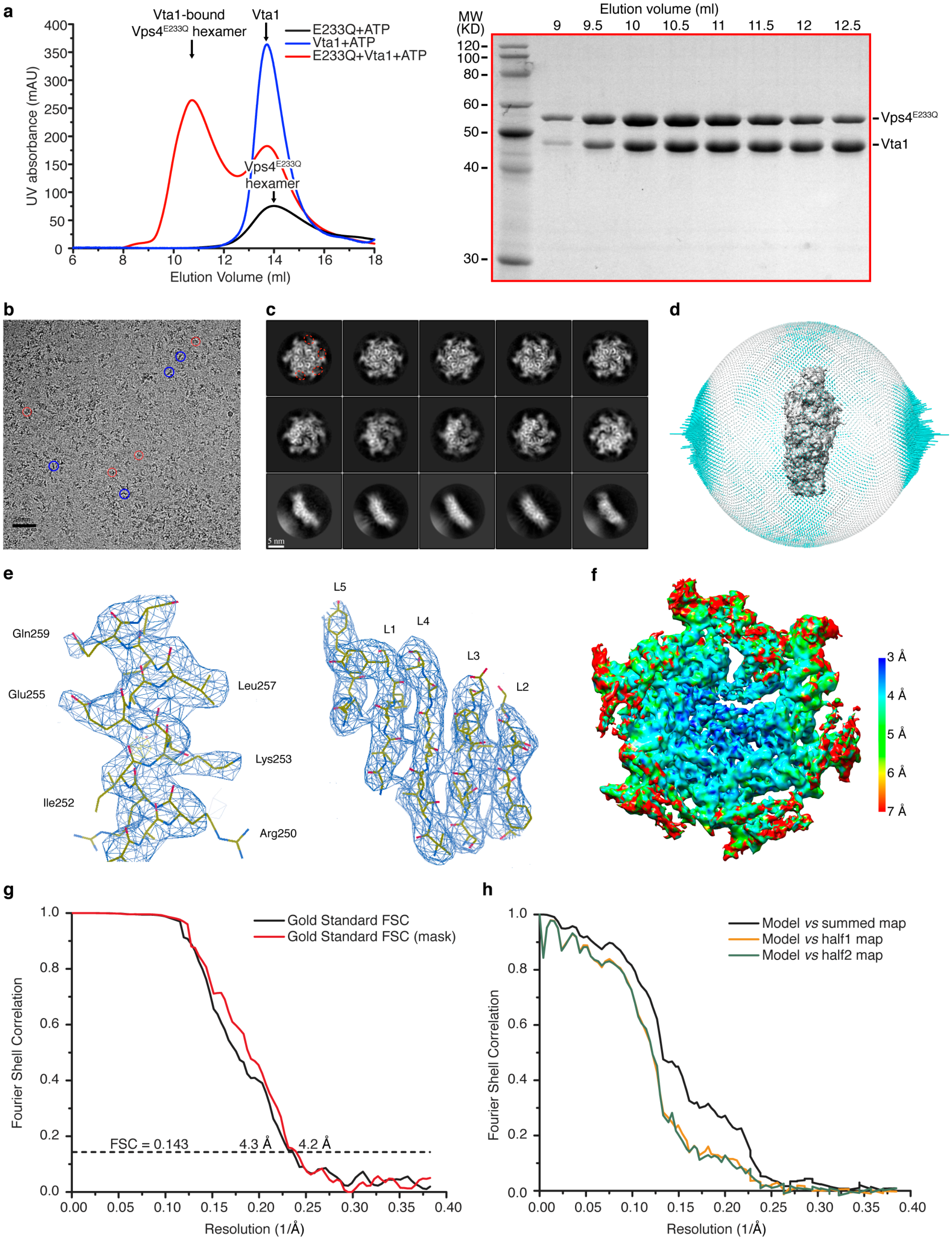

Supplementary Figure 5 Purification and cryo-EM analysis of the ATP-bound Vps4^{E233Q}-Vta1 complex.

(a) SEC of the formation of the ATP-bound Vps4^{E233Q}-Vta1 complex (left panel) and the SDS-PAGE gel (13% resolving gel) of the corresponding fractions from the

chromatogram of the Vps4^{E233Q}-Vta1 mixture in the presence of ATP (right panel). **(b)** A representative electron micrograph of the ATP-bound Vps4^{E233Q}-Vta1 complex low pass filtered to 5 Å. A few typical protein particles of top views and side views are marked by red and blue circles, respectively. **(c)** Typical good reference-free 2D class averages from the single particle images. The additional rod-like densities compared with the 2D class averages of the Vps4^{E233Q} hexamer are highlighted by red dashed circles. **(d)** Euler angle distribution of particles contributing to the final reconstruction. Each cylinder represents one view and the size of the cylinder is proportional to the number of particles for that view. **(e)** Examples of the densities in the cryo-EM map of the ATP-bound Vps4^{E233Q}-Vta1 complex, including an a helix (residues 249-260) and the b sheet (residues 168-172 for strand 1, 193-198 for strand 2, 227-232 for strand 3, 270-276 for strand 4, 293-295 for strand 5) from subunit C. The atomic models are displayed in stick representation, and the density maps were showed as blue mesh. Segmented density maps are displayed at ~ 7 σ contour level. **(f)** The EM density map color-coded to show the local resolution as estimated by ResMap. **(g)** Gold-standard FSC curves of the reconstruction. The reconstruction gives an overall resolution of 4.3 Å, and 4.2 Å after applying a soft mask around the rigid part (excluding the subunit A and all bound Vta1 proteins). **(h)** FSC curves for the cross-validation of the atomic models of the ATP-bound Vps4^{E233Q}-Vta1 complex.

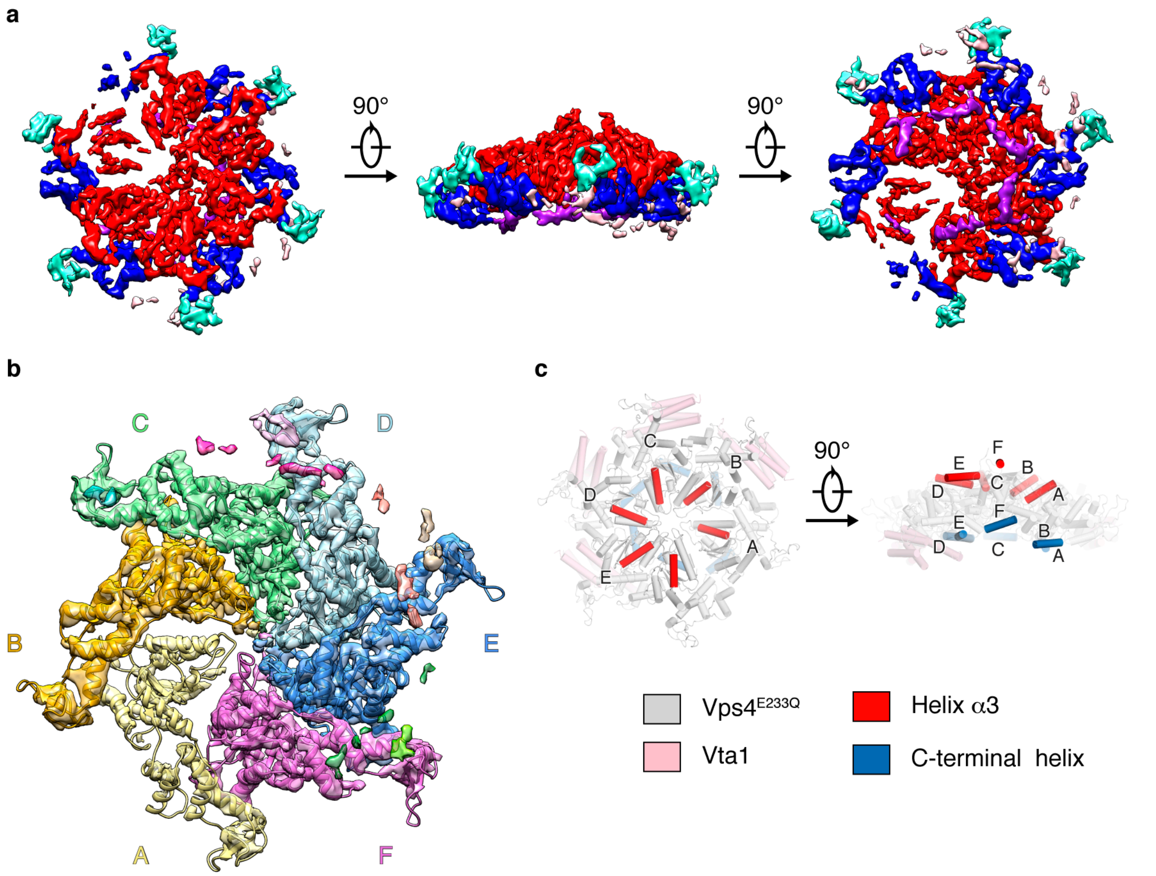

Supplementary Figure 6 Overall structure of the Vps4^{E233Q} hexamers in the ATP-bound Vps4^{E233Q}-Vta1 complex.

(a) Different views of the EM density map of the ATP-bound Vps4^{E233Q}-Vta1 complex at 6.8 σ contour level to show the ATP-bound Vps4^{E233Q} hexamer. The coloring scheme is the same as in Fig. 1d. **(b)** Docking of the atomic model into the cryo-EM density map. The density map is contoured at the 6.8 σ level, and only the models of the Vps4^{E233Q} subunits are shown. The coloring scheme is the same as in Fig. 1e. **(c)** Different views of the helix α3 and C-terminal helix in the Vta1-bound Vps4^{E233Q} hexamer to show the spiral arrangement of Vps4^{E233Q} subunits.

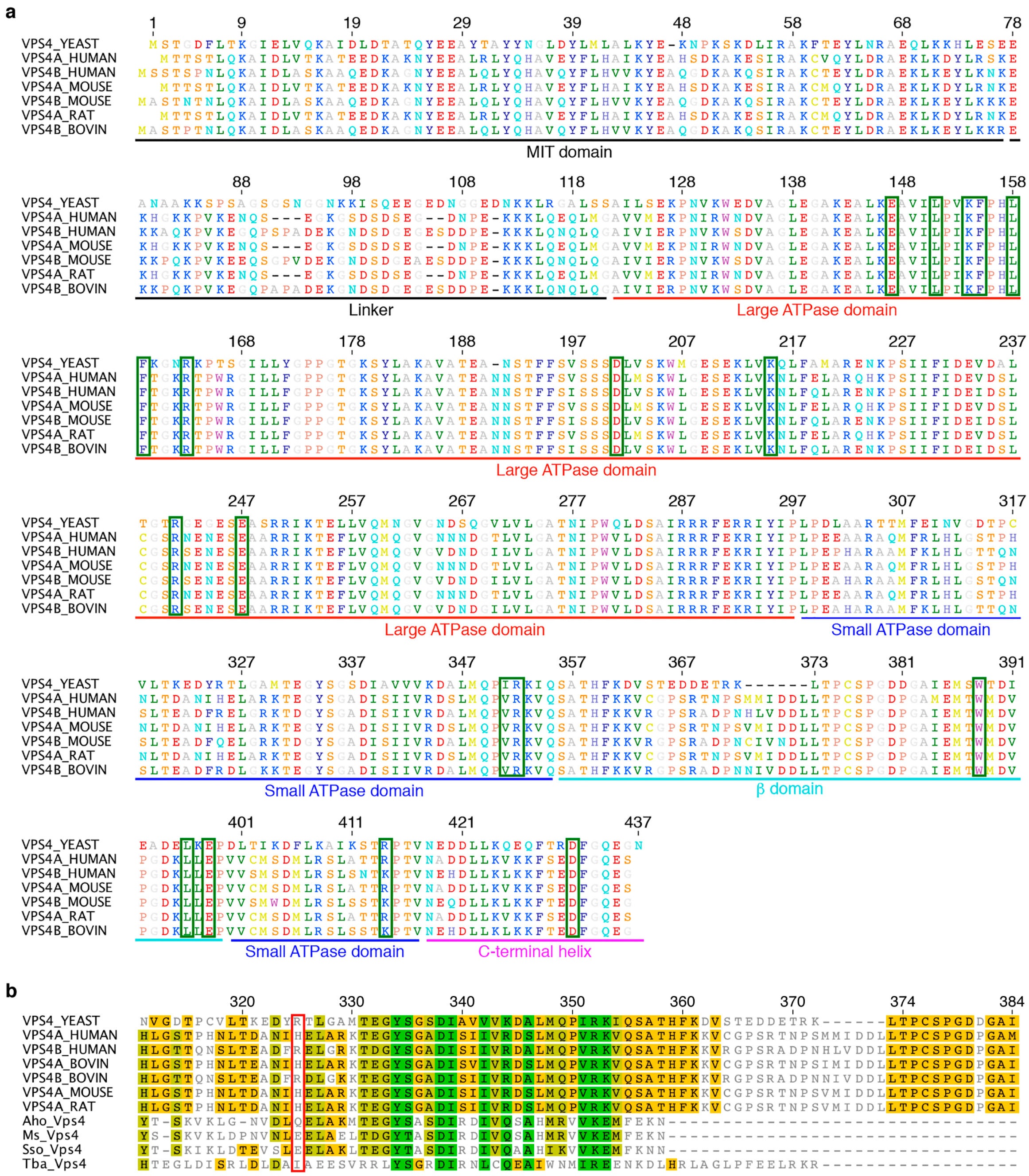

Supplementary Figure 7 Sequence alignment of Vps4 proteins.

(a) Sequence alignment of Vps4 proteins from eukaryotes. The conserved domains are indicated above the sequence alignment. The residues are numbered according to the yeast Vps4 sequence. The residues potentially contributing to intersubunit interactions are highlighted by green boxes. The Uniprot IDs for the aligned sequences are: VPS4_YEAST: P52917; VPS4A_HUMAN: Q9UN37; VPS4B_HUMAN: O75351; VPS4A_MOUSE: Q8VEJ9; VPS4B_MOUSE: P46467; VPS4A_RAT: Q793F9; VPS4B_BOVIN: Q0VD48. **(b)** Sequence alignment of Vps4 proteins from eukaryotes

and crenarcheota. The residues are numbered according to the yeast Vps4 sequence. Only residues around R325 are shown. R325 in yeast sequence and aligned amino acids from other sequences are boxed by red lines. The Uniprot IDs for the aligned sequences are: VPS4_YEAST: P52917; VPS4A_HUMAN: Q9UN37; VPS4B_HUMAN: O75351; VPS4A_BOVIN: G3X8E2; VPS4B_BOVIN: Q0VD48; VPS4A_MOUSE: Q8VEJ9; VPS4A_RAT: Q793F9; Aho_Vps4 (*A. hospitalis*): F4B4B0; Ms_Vps4 (*M. sedula*): A4YHC5; Sso_Vps4 (*S. solfataricus*): Q97ZJ7; Tba_Vps4 (*T. barophilus*): F0LHW6.

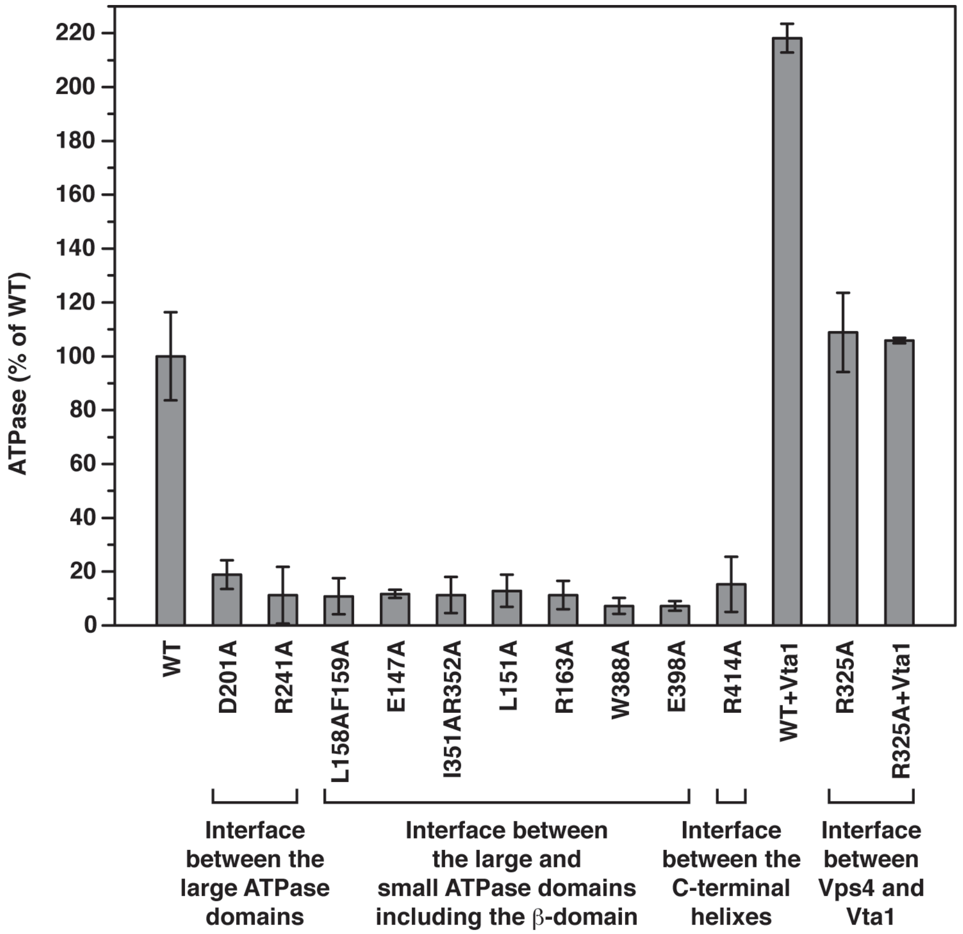

Supplementary Figure 8 ATPase activity assays of the wild-type and mutant Vps4 proteins.

Data are represented as the average of three independent experiments. Error bars represent s.d.

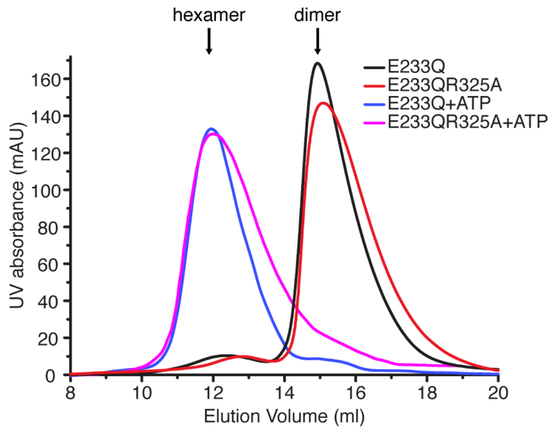

Supplementary Figure 9 Analysis of the oligomeric states of some Vps4^{E233Q} mutants.

SEC analyses of the oligomeric states of the Vps4^{E233Q} mutants E233Q and E233QR325A in the absence or presence of ATP by SEC. Both mutants ran as a dimer without ATP and a hexamer in the presence of ATP.

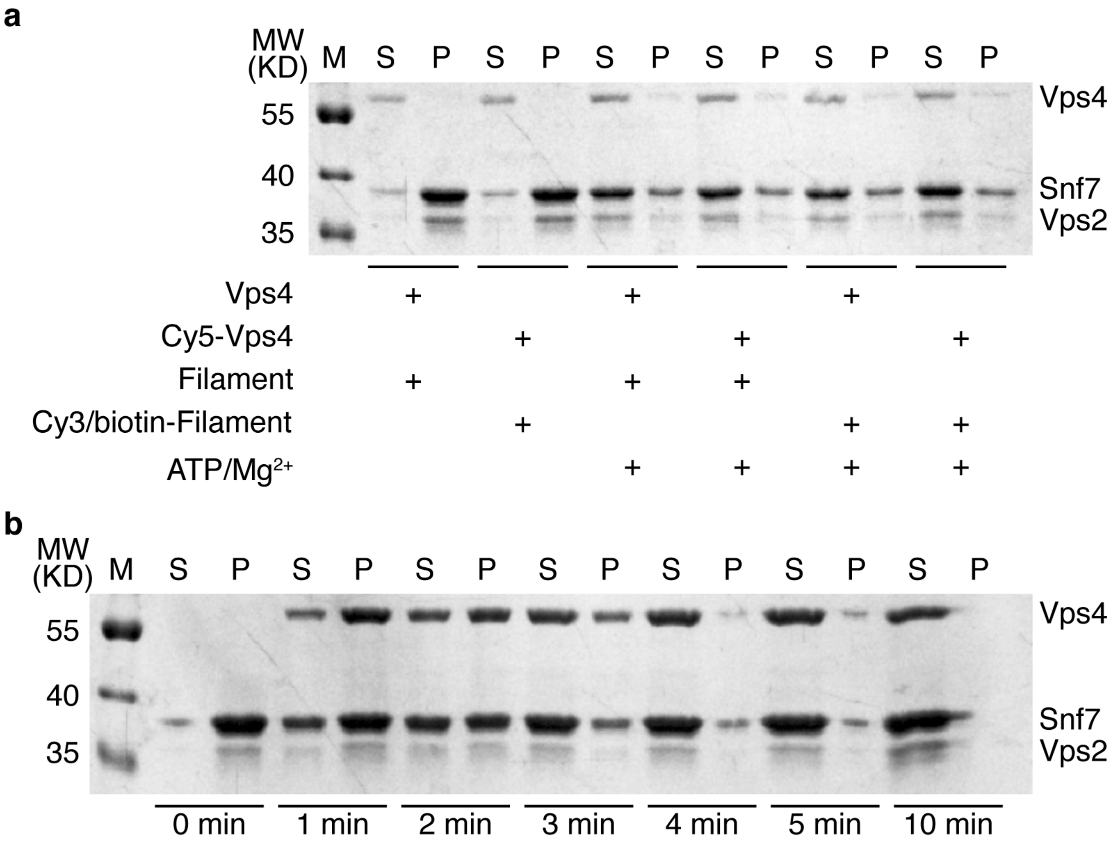

Supplementary Figure 10 Filament disassembly activity assays.

(a) Filament disassembly activity assays of unlabeled and labeled Vps4 towards unlabeled and labeled filaments. **(b)** Filament disassembly activities of Vps4 molecules at selected time points. S, supernatant; P, pellet.

Supplementary Table 1 Data collection and model statistics.

	The ATP-bound Vps4^{E233Q} hexamer	The ATP-bound Vps4 ^{E233Q}-Vta1 complex
**Data collection**		
Electron microscope	FEI Titan Krios	
Voltage (kV)	300	
Electron Detector	Gatan K2 camera	
Pixel size (Å)	1.30654	
Electron dose (e^{-}/Å^{2})	50 (32 frames)	
Defocus range (µm)	1.5~3.0	
**3D Reconstruction**		
Software	RELION 1.3	
Particles for final refinement	106,918	106,106
Symmetry	C1	
Resolution (unmasked, Å)	4.1	4.3
Resolution (masked, Å)	3.9	4.2
Map sharpening B-factor (Å^{2})	-200	-180
**Refinement**		
Model composition		
Peptide chains	6	14
Protein Residues	1,914	2,298
Ligands (ATP)	5	5
R.m.s deviations		
Bonds length (Å)	0.0079	0.0092
Bonds Angle (˚)	1.1655	1.4531
Ramachandran plot statistics (%)		
Favored	88.54	93.35
Allowed	8.99	4.98
Outlier	2.47	1.67
Molprobity score	1.53	1.71
EMRinger score	0.60	0.45

Supplementary Table 2 Filament disassembly rates in the single molecule experiments and the bulk experiment

	Experimental conditions	Disassembly rates (min^{-1})*
Single molecule experiments	1 μM Vps4	0.30 ± 0.01
	1 μM Vps4 + 2 μM Vta1	2.1 ± 0.1
	1 μM Cy5-Vps4 + 2 μM Vta1	1.8 ± 0.1
	100 nM Vps4	0.019 ± 0.003
	100 nM Vps4 + 200 nM Vta1	0.26 ± 0.01
Bulk experiment	1 μM Vps4	0.37 ± 0.05

* Apparent reaction rates were estimated by single or double exponential fitting of time-dependent decay points of reaction components. In the case of double exponential fitting, apparent rates were calculated as the reciprocal of the weighted average lifetime of two exponential populations. Rate was shown as average ± s.e.m.
